# Paleoceanography and dinoflagellate cyst stratigraphy across the Lower–Middle Pleistocene Subseries (Calabrian–Chibanian Stage) boundary at the Chiba composite section, Japan

**DOI:** 10.1186/s40645-021-00438-3

**Published:** 2021-09-01

**Authors:** Eseroghene J. Balota, Martin J. Head, Makoto Okada, Yusuke Suganuma, Yuki Haneda

**Affiliations:** 1 Department of Earth Sciences, Brock University, St. Catharines, ON L2S 3A1 Canada; 2 Department of Earth Sciences, Ibaraki University, 2-2-1 Bunkyo, Mito, Ibaraki, 310-8512 Japan; 3 National Institute of Polar Research, 10-3 Midori-cho, Tachikawa, Tokyo, 190-8518 Japan; 4 Department of Polar Science, School of Multidisciplinary Sciences, The Graduate University for Advanced Studies (SOKENDAI), Hayama, Japan; 5 Geological Survey of Japan, AIST, Higashi 1-1-1 Central 7, Tsukuba, Ibaraki, 305-8567 Japan

**Keywords:** Dinoflagellate cysts, Paleontology, Palynology, Paleoceanography, MIS 19, GSSP, Pleistocene, Chiba, Chibanian, Japan

## Abstract

A dinoflagellate cyst record from the highly resolved Chiba composite section in Japan has been used to reconstruct sea-surface paleoceanographic changes across the Lower–Middle Pleistocene Subseries (Calabrian–Chibanian Stage) boundary at the global stratotype, constituting the first detailed study of this microfossil group from the Pleistocene of the Japanese Pacific margin. Cold, subarctic water masses from 794.2 ka gave way to warming and rapid retreat of the Subpolar Front at 789.3 ka, ~ 2000 years before the end of Marine Isotope Stage (MIS) 20. Throughout the fully interglacial conditions of MIS 19c, assemblages are consistent with warm sea surface temperatures but also reveal instability and latitudinal shifts in the Kuroshio Extension system. The abrupt dominance of *Protoceratium reticulatum* cysts between 772.9 and 770.4 ka (MIS 19b) registers the influence of cooler, mixed, nutrient-rich waters of the Kuroshio–Oyashio Interfrontal Zone resulting from a southward shift of the Kuroshio Extension. Its onset at 772.9 ka serves as a local ecostratigraphic marker for the Chibanian Stage Global Boundary Stratotype Section and Point (GSSP) which occurs just 1.15 m (= 1300 years) below it. An interval from 770.1 ka to the top of the examined succession at 765.8 ka (MIS 19a) represents warm, presumably stratified but still nutrient-elevated surface waters, indicating a northward shift of the Kuroshio Extension ~ 5 kyrs after the termination of full interglacial conditions on land.

**Supplementary Information:**

The online version contains supplementary material available at 10.1186/s40645-021-00438-3.

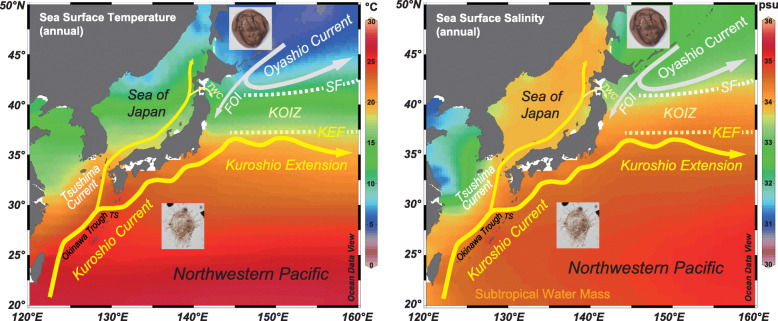

## Introduction

**Fig. 1:**
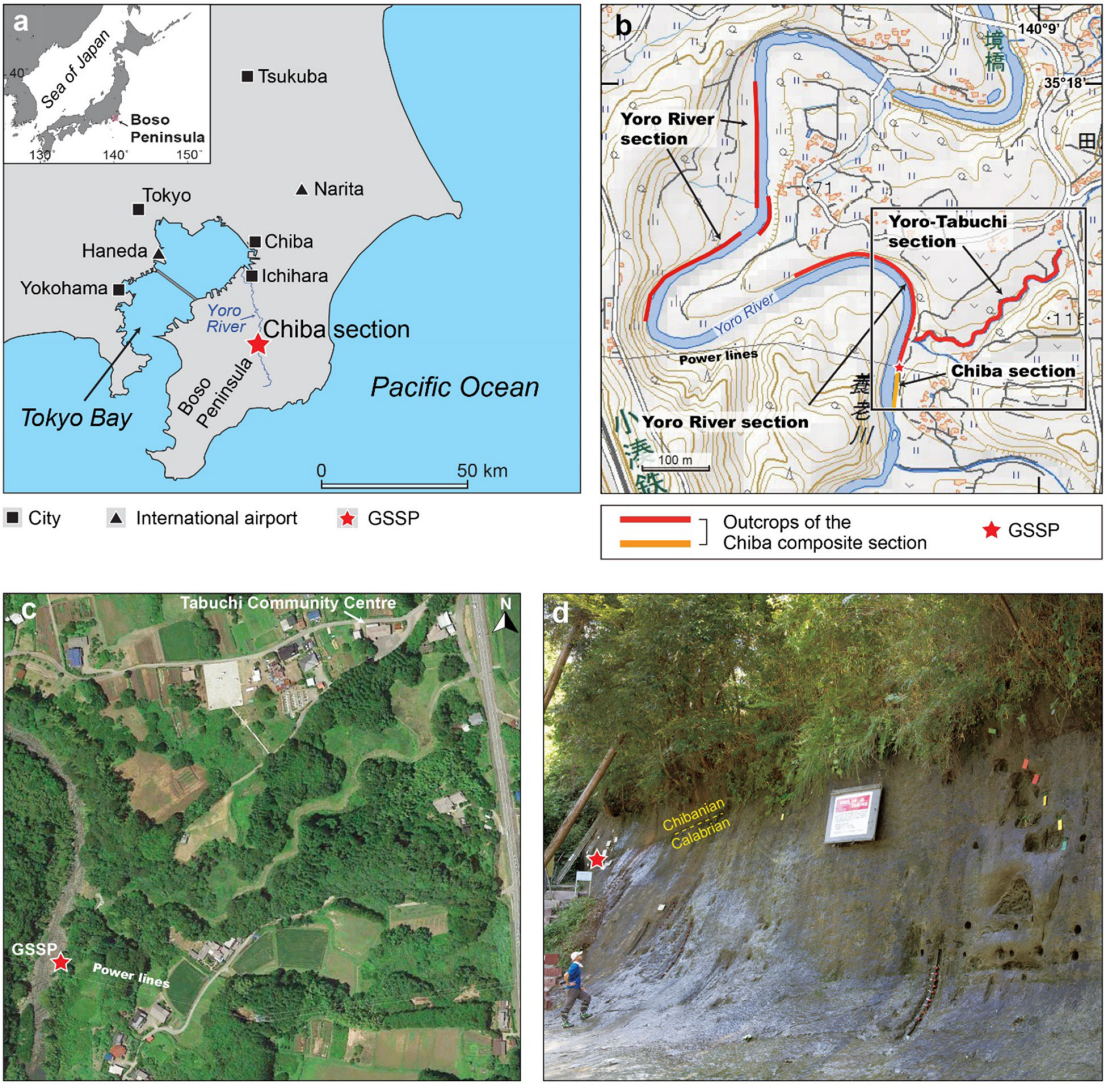
Location of the Chiba and Yoro-Tabuchi sections examined in the present study. **a** Location of the Chiba section in the central Boso Peninsula. Selected cities and the two nearby international airports of Haneda (Tokyo International Airport) and Narita (New Tokyo International Airport) are shown. **b** Topography of the Yoro River in the vicinity of the Chiba and Yoro-Tabuchi sections. The Chiba section forms part of the Yoro River section, and is continuous with the Yoro-Tabuchi section. All are part of the Chiba composite section. The Global Boundary Stratotype Section and Point (GSSP) is located on the east bank of the Yoro River near where the power lines cross overhead (from fig. 8c of Suganuma et al., in press). **c** Google Earth image of the square shown in (**b**). **d** Chiba section showing the position of the GSSP (red star) which defines the boundary between the Calabrian and Chibanian stages and Lower and Middle Pleistocene subseries (from fig. 10a of Suganuma et al., in press)

The Chiba section, located in the central Boso Peninsula, Chiba Prefecture, east-central Japan, serves as the Global Boundary Stratotype Section and Point (GSSP) for the Chibanian Stage and Middle Pleistocene Subseries (Head 2019, 2021; Suganuma et al., in press). It is one of five sections comprising the Chiba composite section, an expanded and continuous silty marine sedimentary succession that bears excellent records of paleoceanographic and paleoclimatic change across the Early–Middle Pleistocene boundary. The Chiba section (35° 17′ 39.6′′ N, 140° 08′ 47.6′′ E to 35° 17′ 36.9′′ N, 140° 08′ 47.2′′ E) is a segment of the Yoro River exposure and is located 70 m west the Yoro-Tabuchi section (35° 17′ 48.1′′ N, 140° 09′ 02.1′′ E to 35° 17′ 41.1′′ N, 140° 08′ 49.7′′ E) which extends the composite section upwards (Fig. 1). The Matuyama–Brunhes paleomagnetic reversal, which falls within Marine Isotope Stage (MIS) 19, represents the primary guide to the Early–Middle Pleistocene boundary (Head et al. 2008). The Chiba and Yoro-Tabuchi sections together yield one of the most detailed records available through MIS 19 and the Matuyama–Brunhes paleomagnetic reversal (Head 2021).

The Chiba composite section has been the subject of numerous micropaleontological studies, mostly involving benthic and planktonic foraminifera, radiolarians, calcareous nannofossils, and pollen (Kazaoka et al. 2015; Suganuma et al. 2018; Kameo et al. 2020). Dinoflagellate cysts have not previously been published from the Chiba composite section or indeed at high temporal resolution from any Pleistocene locality along the Pacific margin of Japan. Dinoflagellates are protists and include phototrophic, heterotrophic, and mixotrophic species (Schnepf and Elbrächter 1992). It is likely that most, if not all, phototrophic (chloroplast-bearing) dinoflagellates are also capable of ingesting prey and are hence mixotrophic (Jeong et al. 2010). Dinoflagellates represent a significant component of the modern plankton in the world’s oceans. The resting stage, known as a cyst, is usually the only part of the life cycle that is geologically preservable. For species that produce organic-walled cysts, the outer wall withstands acid treatments and is studied in palynological preparations. Only ~ 13–16% of the ~ 2000 dinoflagellate species living today produce organic-walled cysts (Head 1996a; Penaud et al. 2018), but the modern distributions of these cysts on the ocean floor demonstrate their sensitivity to a variety of sea-surface parameters including temperature, salinity, and nutrient levels. They are therefore used widely to reconstruct past oceanographic conditions (de Vernal et al. 2020).

Phototrophic dinoflagellates are confined to the photic zone, and the heterotrophic dinoflagellates will also presumably be concentrated here where their prey is plentiful. The record of dinoflagellate cysts in sedimentary archives is therefore generally taken to represent sea-surface conditions. Interpreting seasonal signals from the dinoflagellate cyst record is, however, more uncertain. Cyst-producing dinoflagellates in high northern latitudes bloom from spring through fall during a short biological summer, and are dormant as resting cysts during the winter months (Matthiessen et al. 2005). Nonetheless, sediment trap studies undertaken in lower latitudes have shown that cyst production can occur at various time of the year, with different species producing cysts at different times. A sediment trap study from the Cariaco Basin off Venezuela linked cyst production to times of seasonal, wind-driven upwelling, with both heterotrophs and phototrophs having greatest fluxes during February through April and also to a lesser extent in July (Bringué et al. 2019). A study from southwest Taiwan waters in the South China Sea, which is influenced by both the winter (March–April) and summer (July–August) East Asian monsoons, showed increases in the fluxes of almost all taxa in July–August, reflecting increased river and nutrient input during the summer monsoon (Li et al. 2018). In two embayments along the southern coast of South Korea, phototrophic cyst production was also found to be mostly restricted to the warmest months (July–September) and the fall (Shin et al. 2012, 2018). Within the water column, motile cells of *Gonyaulax spinifera*, which produce cysts assignable to the cyst-defined genus *Spiniferites*, were found to be largely restricted to the months of March–August based on a 5-year survey in the Yellow Sea of China (Liu et al. 2017). At Omura Bay, southwestern Japan, cyst fluxes of heterotrophic species were found to be greatest in fall and winter, correlating with the production of diatoms upon which they feed, whereas autotrophs occurred more generally through the year (Fujii and Matsuoka 2006) but with *Spiniferites* species having greatest fluxes in the late spring and fall (Pospelova et al. 2018). Pospelova et al. (2018) compiled the results of eight globally distributed sediment trap studies, focusing on fluxes of the diverse and widespread phototrophic genus *Spiniferites*. These authors found that although the cyst fluxes of all species responded in similar ways, they were not driven by a uniform seasonal pattern. Local changes in hydrography instead appeared to be the main triggers, with elevated cyst production corresponding to conditions reflecting minimal turbidity, availability of nutrients, some water column stratification or stability, and an absence of sea-ice (Pospelova et al. 2018). Significantly for the present study, motile cells of *Protoceratium reticulatum*, another important cyst-producing species, were found to be present only during the summer months in the surface waters off northeastern Japan (Koike et al. 2006, and see below). It therefore appears that the Chiba dinoflagellate cyst record reflects spring–fall sea-surface conditions at least for some species, including the cysts of *Protoceratium reticulatum*. It is possible or even likely, however, that the heterotrophs represent cooler-season conditions. More studies of the monthly production of motile cells and cysts in the surface waters off eastern Japan are needed to gain clearer insights into the seasonal signal carried by the Chiba dinoflagellate cyst record.

**Fig. 2:**
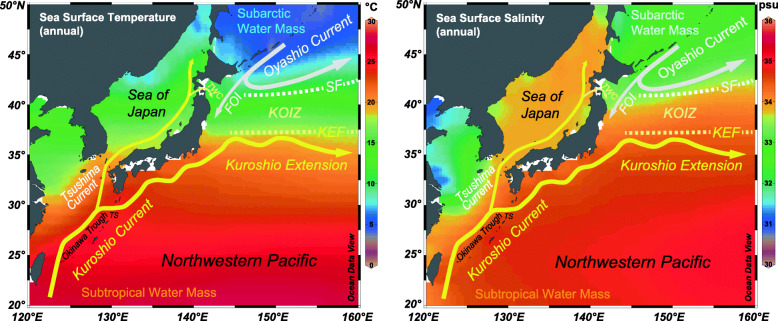
Present oceanography of the northwestern Pacific Ocean, showing annual sea-surface temperature and salinity from the World Ocean Atlas 2013 (Locarnini et al. 2013) drawn using Ocean Data View software (Schlitzer 2015). Major oceanographic features are included. TWC = Tsugaru Warm Current. FOI = first Oyashio intrusion. SF = Subarctic Front formed by the Subarctic Current, an eastward extension of the Oyashio Current. KEF = Kuroshio Extension Front. KOIZ = Kuroshio–Oyashio Interfrontal Zone, a zone of mixing between the SF and KEF. TS = Tokara Strait. Adapted from fig. 1 of Suganuma et al. (2018)

Two dominant surface current systems meet today off the Boso Peninsula: the warm and nutrient-depleted Kuroshio Current flowing from the south, and the cold nutrient-rich subarctic Oyashio Current flowing from the north. The Subarctic Front forms where the cold Oyashio Current meets and descends beneath the Kuroshio Current. These currents mix to form a significant latitudinal sea-surface temperature (SST) gradient that is most pronounced off the Boso Peninsula (Fig. 2). These two currents have been interacting since at least the Pleistocene (Okazaki et al. 2010; Gallagher et al. 2015) and their respective strengths and positions reflect broader changes in the ocean–atmosphere system.

The present study aims to document the organic-walled dinoflagellate cyst record of the Chiba composite section, and using modern species distributions reconstruct its paleoceanographic evolution from late MIS 20 to mid-MIS 19a. This study revises and expands upon an earlier unpublished study of the dinoflagellate cysts from the Chiba composite section (Balota 2018). MIS 19c serves as an analogue for the present pre-industrial interglacial, both having similar orbital configurations (e.g., Tzedakis et al. 2012; Yin and Berger 2012; Giaccio et al. 2015; Vavrus et al. 2018; Nomade et al. 2019). The Chiba composite section is therefore optimally positioned to capture the same interplay between the Kuroshio and Oyashio currents that is now observed off the Boso Peninsula (Suganuma et al. 2018).

### Chiba composite section

The Chiba composite section has been studied intensively and yields one of the most highly resolved marine records of the Matuyama–Brunhes paleomagnetic reversal available (Suganuma et al. 2015; Okada et al. 2017; Simon et al. 2019; Haneda et al. 2020a); this boundary having already been chosen as the primary guide to the GSSP (Head et al. 2008). The succession is astronomically dated both by high-resolution foraminiferal δ^18^O stratigraphy (Okada et al. 2017; Suganuma et al. 2018; Haneda et al. 2020b) and a zircon U-Pb age of 772.7 ± 7.2 ka (Suganuma et al. 2015) obtained from the ~ 2-cm-thick Ontake-Byakubi-E (Byk-E) tephra bed (Takeshita et al. 2016) which provides a local and regional marker for the GSSP. The GSSP is dated astronomically at 774.1 ka and occurs just below the top of Marine Isotope Substage (MIS) 19c (Suganuma et al. 2018; Suganuma et al., in press). The directional midpoint of the Matuyama–Brunhes boundary occurs 1.1 m above the GSSP and has an astronomical age of 772.9 ka (Suganuma et al. 2018; Haneda et al. 2020a).

The Chiba composite section occurs within in the upper part of the Kokumoto Formation, Kazusa Group, and represents continuous and generally stable deposition on the continental slope of the eastern (Pacific) margin of Japan. Trace fossil evidence suggests water depths exceeding 800 to 1000 m (Nishida et al. 2016). Along with high sedimentation rates of 89 cm/kyr at the boundary, with no evidence of abrupt deposition, this open-ocean setting allows the integration of both marine and terrestrial paleoenvironmental proxies (Suganuma et al. 2018). High-resolution benthic and planktonic foraminifera δ^18^O and pollen records extend from 801 ka in MIS 20 to 748 ka (752 ka for the pollen) in MIS 18. All three substages of MIS 19 are recognized in the benthic foraminiferal δ^18^O record (Haneda et al. 2020b). Following the substage characterization of Nomade et al. (2019), MIS 19c corresponds to the onset (Termination IX) and warmest part of the stage (the MIS 19 plateau), MIS 19b to the first climatic cooling, and MIS 19a to the most unstable part of Stage 19. The current Chiba composite section age model is based on Suganuma et al. (2018) but uses the full benthic foraminiferal δ^18^O benthic dataset of Haneda et al. (2020b) to identify the positions of stage and substage boundaries as follows: 787.5 ka (MIS 20–19c), 773.9 ka (MIS 19c–19b), 770.1 ka (MIS 19b–19a), and 756.9 ka (MIS 19a–18) with a chronological uncertainty of ~5 kyr (Haneda et al. 2020b; Suganuma et al., in press). In addition, a significant sea surface and subsurface temperature drop just prior to the onset of MIS 19 seems to represent a Younger Dryas-type cooling event (Suganuma et al. 2018). The terrestrial climate as reflected in the pollen record largely mirrors these changes, with full interglacial conditions extending from 785.0 to 775.1 ± 5.0 ka with a duration of 9.9 kyr and so mostly coinciding with MIS 19c (Suganuma et al. 2018, in press; Kameo et al. 2020; Kubota et al. 2021).

Additional marine paleoenvironmental proxies obtained from the Chiba composite section, although at lower stratigraphic resolution, include calcareous nannofossils, planktonic and benthic foraminifera, radiolaria, and Mg/Ca subsurface (> 100 m) winter–spring paleotemperature records from the planktonic foraminifera *Globorotalia inflata* (Suganuma et al. 2018).

### Modern oceanographic setting

The waters off the Boso Peninsula are presently under the influence of the subtropical Kuroshio Current, the subarctic Oyashio Current, and the Tsugaru Warm Current. The Oyashio Current descends beneath the Kuroshio Current to form the Subarctic Front in the northwestern Pacific Ocean. A pronounced latitudinal sea-surface temperature (SST) gradient which is greatest off the Boso Peninsula is created by the mixing of these two currents (Fig. 2).

The Kuroshio Current is a western boundary current that forms part of the wind-driven North Pacific Subtropical Gyre. It transports heat and salt from the Indo-Pacific Warm Pool into the higher latitudes of the North Pacific. From its inception east of the Philippines, it flows northeast past Taiwan, into the East China Sea where it follows the Okinawa Trough, through the Tokara Strait, and then eastwards along the south coast of Japan where it follows a straight or meandering path (interannual bimodality) depending on the magnitude of upstream transport (Qiu 2019). Off the Boso Peninsula at around 35° N, the current diverts eastwards away from the coast of Japan and forms the Kuroshio Extension system. Between 140° E and 160° E, the mean axis of the Kuroshio Extension is displaced interannually between 33.5° N and 35.5° N (Qiu 2019). The Kuroshio Current off central Japan today has an annual mean sea surface temperature (SST) and salinity (SSS) of 19–21 °C and 34 psu, respectively (Locarnini et al. 2013; Zweng et al. 2013). Southward latitudinal SST and SSS gradients across the Kuroshio Extension are + 0.7 ± 0.2 (in August) to + 1.8 ± 0.7 (in December) °C/100 km and − 0.10 (in March) to − 0.19 (in September) psu/100 km, respectively (Kida et al. 2015). The surface waters of the Kuroshio Current for most of the year are oligotrophic (*kuro* and *shio* in Japanese mean “dark current” with reference to the clear dark-blue waters lacking phytoplankton and detritus), although some nutrient mixing occurs toward the end of winter for a few months. A nutrient stream flows below the surface euphotic zone and is separated from it by a strong pycnocline. Nonetheless, some transfer of nutrients to the euphotic zone occurs, particularly downstream (Komatsu and Hiroe 2019; Nagai et al. 2019), and partly explains the rich pelagic fish stock occurring in the Kuroshio Current (Saito 2019). This nutrient stream contributes significantly to the Kuroshio–Oyashio Interfrontal Zone (KOIZ) (Komatsu and Hiroe 2019), where the Kuroshio Current mixes with nutrient-rich waters of the Oyashio Current in the region off central Japan today to create the strongest latitudinal sea-surface temperature (SST) gradient in the northwestern Pacific Ocean.

The Oyashio Current is also a western boundary current although within the wind-driven North Pacific Subpolar Gyre (Qiu 2019). Its source area, the subarctic North Pacific, is dominated by upwelling and excessive precipitation over evaporation, making this a cold, low-salinity, nutrient rich current. It is an extension of the East Kamchatka Current but receives input from the Sea of Okhotsk which changes its properties so that elevated dissolved-oxygen values occur throughout its upper 700 m depth (Qiu 2019). Its name, *Oya* and *shio* in Japanese meaning “parent current,” refers to the high biological productivity it sustains. The Oyashio Current flows along the east coast of Hokkaido and then bifurcates, with one branch deflecting east-northeastwards as the Subarctic Current. The southern margin of this current meets warm saline waters from the south to generate the Subarctic Front which has temperature and salinity values of 5°C and 33.8 psu at a depth of 100 m. The other branch flows southward along the east coast of Honshu as the first Oyashio intrusion and can be defined by the 5 °C isotherm at 100 m depth. Its southward penetration is influenced by the seasonally-fluctuating Aleutian Low pressure system and presently varies between 38.5° N in April and 41.5° N in December, but also varies on interannual and longer timescales (Qiu 2019). The first Oyashio intrusion potentially has a direct impact on the continental shelf and slope off the Boso Peninsula. Today off northern Japan at 42.5–43.5° N, the Oyashio Current has a modern annual mean SST and SSS of 6–8 °C and 33.0 psu, respectively (Locarnini et al. 2013; Zweng et al. 2013). Southward latitudinal SST and SSS gradients across the Subarctic Current are from + 2.0 ± 1.5 (in October) to + 3.3 ± 0.4 (in April) °C/100 km, and − 0.35 (in April and December) to − 0.54 (in September) psu/100 km, respectively (Kida et al. 2015).

In addition to the Kuroshio and Oyashio currents, the Tsugaru Warm Current influences the waters off the Boso Peninsula. The Tsugaru Warm Current is an extension of the Tsushima Current, itself a branch of the Kuroshio Current, and flows from the Sea of Japan through the Tsugaru Strait. It then flows southward along the eastern coast of Honshu, and landward of the first Oyashio intrusion. It contrasts with Oyashio waters in having warmer temperatures (greater than ~ 6 °C) and relatively high salinities (33.7–34.2) (Hanawa and Mitsudera 1986).

The latitudinal displacement of oceanic fronts in the modern North Pacific, including the subpolar front, derives from changes in the position and intensity of prevailing westerly winds. These changes appear to be linked to the decadal variability of the Aleutian Low, a low-pressure atmospheric system that develops around the Aleutian Island in the North Pacific during winter. When the Aleutian Low intensifies (weakens), it causes a southward (northward) shift in the subpolar front (Taguchi et al. 2012). This relationship has been used to explain variations in winter SST reconstructions through the Chiba composite section (Suganuma et al. 2018; Haneda et al. 2020b; Kubota et al. 2021).

### Previous mid-Pleistocene dinoflagellate cyst studies along the Pacific margin of Japan

No previous stratigraphically detailed dinoflagellate cyst studies of the mid-Pleistocene from the Pacific margin of the Japanese islands are known. However, Harada (1984) presented a relatively low-resolution record of six cores taken from Osaka Bay, about 470 km west of the Chiba locality. The deposits belong to the Osaka Group and include the Azuki tephra bed which has been correlated with MIS 21 (Kazaoka et al. 2015). These deposits should include the interval represented by the Chiba composite section. Osaka Bay today is more fully under the influence of the Kuroshio Current than the Chiba locality. The dinoflagellate cyst assemblages reflect fluctuating nearshore restricted marine paleoenvironments, as might be expected. Some assemblages are dominated by *Polysphaeridium zoharyi* which today thrives in warm, hypersaline nearshore paleoenvironments (Zonneveld et al. 2013). Harada (1984) reported *Lingulodinium machaerophorum*, the *Spiniferites* group, the cysts of *Protoceratium reticulatum* (as *Operculodinium centrocarpum*), and protoperidiniacean cysts as varying usually between subordinate and co-dominant in abundance, with *Tuberculodinium vancampoae* rare to common in most samples but occasionally dominant. *Hystrichokolpoma* sp. was reported outside of the published counts (Harada 1984). In general, this dinoflagellate cyst record does not compare closely with that of the Chiba composite section.

### Modern North Pacific dinoflagellate cyst distributions

Dinoflagellate cyst distributions in modern sediments of the North Pacific and its adjoining seas are well documented as follows: west coast of Mexico, 15–25° N (Kielt 2006; Vásquez-Bedoya et al. 2008; Limoges et al. 2010); northeastern Pacific margin, 25–43° N (Pospelova et al. 2008) and 40–60° N (Radi and de Vernal 2004); eastern Bering and Chukchi seas (Radi et al. 2001); North Pacific, 64–37° N, including the Gulf of Alaska, Bering Sea, western subarctic gyre, Okhotsk Sea, and central North Pacific both north and south of the Subarctic Front (Bonnet et al. 2012); and the South China Sea, 22–4° N (Kawamura 2002, 2004; Li et al. 2020). These large datasets are mostly concentrated around the continental margins and adjoining seas. They are based on standardized laboratory processing and taxonomic methodologies (de Vernal et al. 2020) that allow their basin-wide integration. In addition, the Yellow Sea and East China Sea, 36–29° N, have also been documented (Cho and Matsuoka 2001), and smaller-scale and coastal studies along the western margin of the North Pacific (e.g., Matsuoka 1976 and references therein, 1985, 1987, 1992 and references therein; Pospelova and Kim 2010 and references therein) also provide valuable although more localized information. For example, the small-scale study of surface samples off Hachinohe, northeastern coast of Honshu by Matsuoka (1976) provides useful information on dinoflagellate cyst assemblages influenced by the Tsugaru Warm Current and Oyashio Current. A small-scale study of Akkeshi Bay and Lake Saroma in Hokkaido, northern Japan, similarly provides important information on the influence of the Oyashio Current in this area (Matsuoka 1987).

Although a gap in these large datasets includes much of offshore eastern Japan today, the database along the northern and western margins of the Pacific is sufficient to allow the characterization of assemblages associated with the warm nutrient-depleted Kuroshio Current and the cold, nutrient-rich low-salinty Oyashio Current. These two currents will have provided the main ecological controls on dinoflagellate cyst assemblages in the Chiba composite section across the Early–Middle Pleistocene boundary, and their influence should therefore be detectable in the Chiba record.

## Materials and methods

### Samples

**Fig. 3:**
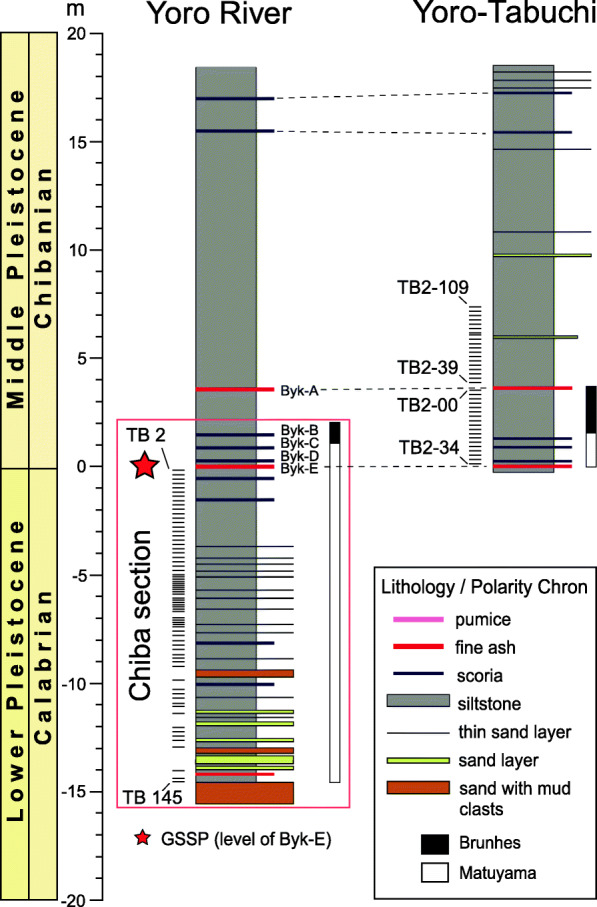
Samples used in the present study. The lithology is from fig. 4 of Suganuma et al. (2018) and paleomagnetic polarity record from Okada et al. (2017) and Haneda et al. (2020a). The vertical scale (in m) represents stratigraphic distance from the Byk-E tephra bed. The GSSP is located at the base of the Byk-E tephra in the Chiba section which is part of the Yoro River section (Fig. 1). The Yoro-Tabuchi section is located about 70 m northeast (Fig. 1) and correlated by tephra beds

In total, 107 samples from late MIS 20 to mid-MIS 19a were processed for marine palynology from the Chiba and Yoro-Tabuchi sections of the Chiba composite section (Fig. 3). Sample numbers are prefixed TB (Chiba section, formerly known as the Tabuchi section) or TB2 (Yoro–Tabuchi section). Samples were collected under the direction of MO and are subsamples of those used for foraminiferal isotope and pollen analysis (Okada et al. 2017; Suganuma et al. 2018; Haneda et al. 2020b).

Seventy samples are from the Chiba section: samples TB145 to TB2 taken from − 14.5 to − 0.15 m (− 12.25 to − 0.15 m excluding sand [turbidite] layers) below the Byk-E tephra bed and representing 794.21–774.31 ka. Thirty-seven samples are from the Yoro-Tabuchi section: samples TB2-34 to TB2-00 and TB2-39 to TB2-109 taken from 0.15 to 7.40 m above the Byk-E tephra bed representing 773.97–765.80 ka. The samples therefore span a total stratigraphic thickness of 19.65 m (excluding turbidite layers) and are dated from 794.21 to 765.80 ka with a duration of 28,400 years using the age model of Suganuma et al. (2018).

Most samples in the present study were taken at 20 cm intervals representing ~ 324 years on average. Some samples within Termination IX, the interval from − 8.5 to − 5.9 m below the Byk-E bed and ranging in age from 791 to 785 ka, are spaced at ~ 10 cm intervals to improve resolution across this important climatic transition.

### Sample processing

Samples were processed at Brock University following standard protocols, as follows. Each sample was dried and weighed. A total of 10 g of sediment was treated with room temperature 7.5% HCl for several hours, and room temperature 38% HF for ~ 2 days with thrice-daily swirling, and neutralization steps using distilled water. No acetolysis, alkali, oxidation, ultrasound, or other treatments were used. One tablet of *Lycopodium clavatum* spores (batch number 1031 containing on average 20,848 spores per tablet; Department of Geology, Lund University, Sweden) was added to the organic residue, which was vigorously mixed and then sieved through a 10-μm nylon mesh. Tablets containing *Lycopodium clavatum* spores are introduced to residues for the purpose of estimating palynomorph concentrations (see below). The spores are darkened by acetolysis during the manufacturing process, and this appears also to increase their propensity to take up stain. These introduced spores cannot then be confused with any in-situ *Lycopodium clavatum* spores that may occur naturally in the samples.

Sieved residues were stained with safranin-o and mounted on microscope slides using glycerine jelly following an adaptation of the method described by Evitt (1984). No oxidation, alkali or ultrasound treatments were applied. At least two slides were made from the > 10 μm sieved residue of each sample for counting under the microscope. The residues from selected samples were also sieved at 20 μm and microscope slides made for morphologic/taxonomic analyses. In addition, surplus residues from samples TB2-22, TB2-00, TB2-97 were heated to ~ 80 °C in a 3% wt. solution of sodium hexametaphosphate for 1 h, ultrasonicated for 1 min, and sieved at 10 μm and 20 μm. This treatment, which removed some of the amorphous organic material and concentrated the cysts, did not noticeably affect the cyst morphology. These samples were used primarily for morphological studies, and the resulting microscope slides bear the suffix MJH. All surplus residues were stored in vials containing glycerine to which one drop of liquefied phenol had been added to prevent microbial growth.

### Microscopy

Microscope slides were examined under a Leica DM 2500 transmitted light microscope by EB, with routine counting performed on the 10-μm slides using a dry 40× objective lens. A 100× oil immersion objective lens was used for detailed morphological analysis and critical identification of small palynomorphs. Dinoflagellate cysts and acritarchs were counted to the highest taxonomic level possible (see below). Benthic microforaminiferal linings were counted only when containing at least six chambers to avoid over-representation owing to fragmentation (Traverse and Ginsburg 1966). Most samples were counted until at least 300 in-situ dinoflagellate cysts had been enumerated. For 16 samples with low dinoflagellate cyst concentrations, at least 200 specimens were counted; and nine samples yielded fewer than 200 counts, even after scanning up to three slides for each. Most samples yielded counts of at least 100 *Lycopodium clavatum* spores.

**Fig. 4:**
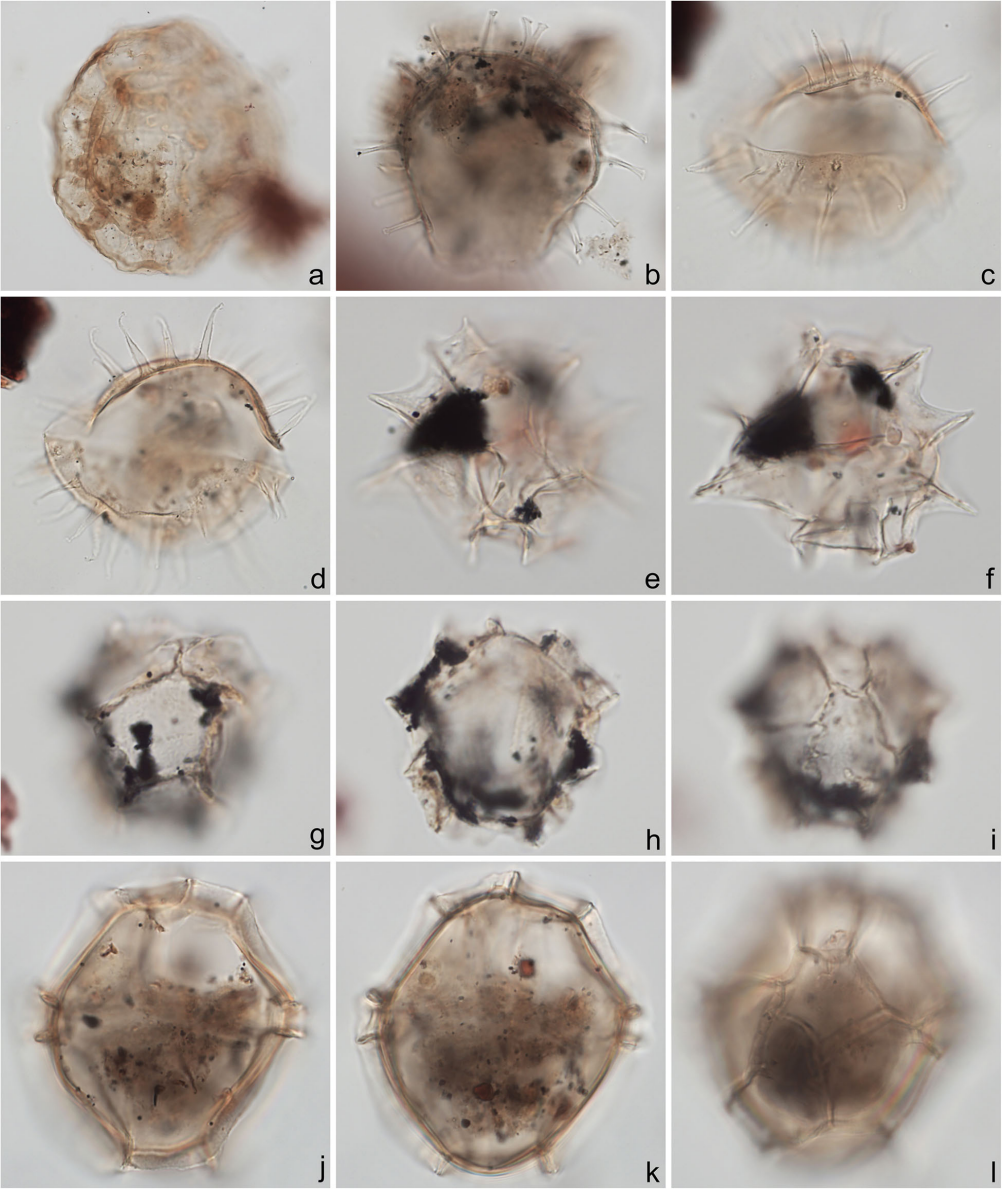
Dinoflagellate cysts from the Chiba composite section. Max. dia. = maximum diameter. EF = England Finder reference. **a** *Tuberculodinium vancampoae*, polar view at upper focus; cyst max. dia. 122 μm; sample TB 16, slide 1 (20 μm), EF E25/4. **b** *Polysphaeridium zoharyi*, apical view of hypocyst at upper focus; central body max. dia. 52 μm; sample TB 98, slide 1 (10 μm), EF E20/1. **c**, **d** *Lingulodinium machaerophorum*, dorsal view at upper and mid-foci; central body max. dia. 58 μm; sample TB2 99, slide 1, EF E36/0. **e**, **f** *Impagidinium aculeatum*, uncertain view at upper- and mid-foci; central body max. dia. 35 μm; sample TB2 26, slide 1, EF E20/3. **g**–**i** *Impagidinium paradoxum*, antapico-dorsal view at upper, mid-, and lower foci; central body length 26 μm; sample TB2 30, slide 2, EF K26/3. **j**–**l** *Impagidinium patulum*, dorsal view at upper, mid-, and lower foci; central body max. length 63 μm; sample TB 92, slide 1, EF Q34/4

A 10-μm slide for each sample was also examined under a 40× objective lens by MJH to estimate qualitatively the relative abundance of terrestrial (mostly woody and cuticular plant tissues) versus marine (amorphous organic matter: AOM) components of the organic residue (Fig. 14g, h). These two broad categories were labeled as dominant, abundant, common, moderate, rare, or trace, to provide a first-order approximation of the palynofacies. The dominant component of each sample is shown in Figs. 16, 18c, and 19h. AOM is a common constituent of marine sediment. Its relatively structureless appearance owes to the unicellular algae that dominate marine biomass and for which resistant structural tissues are often unnecessary (Tyson 1995). AOM can also result from the degradation of terrestrial plant matter, although typically in such circumstances some original structure remains and particles would be present showing a transition between weakly and strongly degraded plant matter. In the Chiba section, the AOM is well preserved and “fluffy” in texture with abundant minute inclusions (Batten 1996), and no features suggesting a terrestrial source were observed. Marine dinoflagellate cysts were sometimes enclosed in this AOM (e.g., Fig. 8d, e, g–i) and it was also occasionally found within the central bodies of the cysts themselves (e.g., Fig. 4j–l); this intimate association supporting the interpretation of a marine origin for the AOM.

Specimens were photographed under bright field illumination by MJH using a Leica DMR microscope equipped with a Leica DFC550 digital camera, and are illustrated in Figs. 4, 5, 6, 7, 8, 9, 10, 11, 12, 13, and 14.

**Fig. 5:**
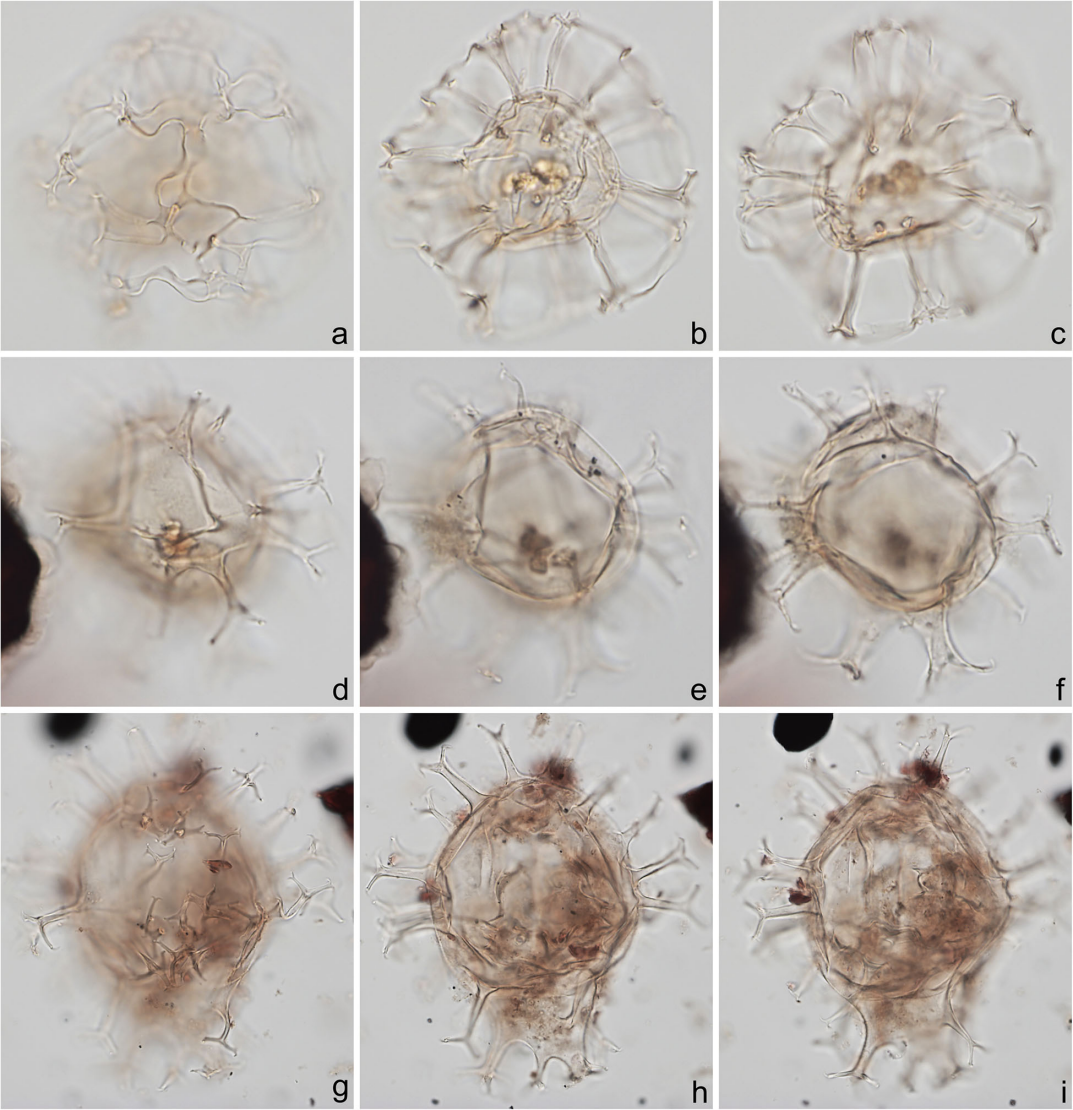
Dinoflagellate cysts from the Chiba composite section. Max. dia. = maximum diameter. EF = England Finder reference. **a**–**c** *Nematosphaeropsis labyrinthus*, equatorial view at upper, mid-, and lower foci showing nearly smooth central body surface; ectophragm max. dia. 52 μm, central body max. dia. 27 μm; sample TB2 97, slide 2 (10 μm) MJH, EF J17/2. **d**–**f** *Spiniferites* cf. *belerius*, dorsal view at upper, mid-, and slightly lower foci; central body length 31 μm; sample TB2 97, slide 3 (20 μm) MJH, EF T21/3. **g**–**i** *Spiniferites mirabilis*, equatorial view at upper, mid-, and lower foci; central body length 62 μm; sample TB 16, slide 1 (10 μm), EF V17/4

**Fig 6:**
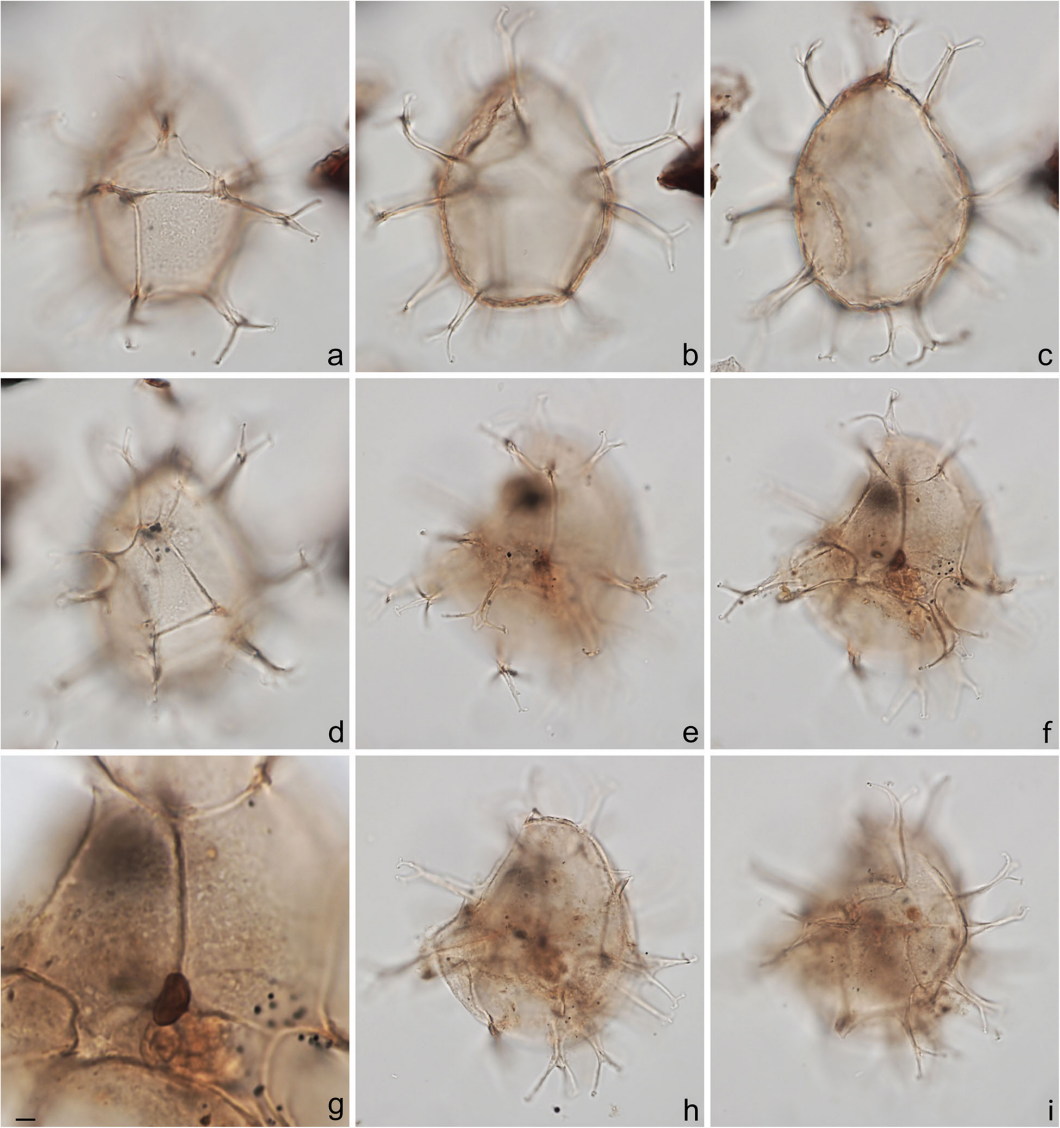
Dinoflagellate cysts from the Chiba composite section. Max. dia. = maximum diameter. EF = England Finder reference. **a**–**i** *Spiniferites pachydermus* sensu Mertens et al. (2015) showing distinctive irregularly perforate surface ornament; **a**–**d** left lateral view at upper to lower foci; central body length 49 μm; sample TB2 00, slide 1 (10 μm), EF D11/2; **e**–**i** right lateral view at upper through lower foci; note irregular surface perforations in **i**; central body length 57 μm; dark ovoidal body in lower center is 5.8 μm long; sample TB 16, slide 1 (20 μm), EF E26/1. Scale bar on **g** = 2 μm

**Fig. 7:**
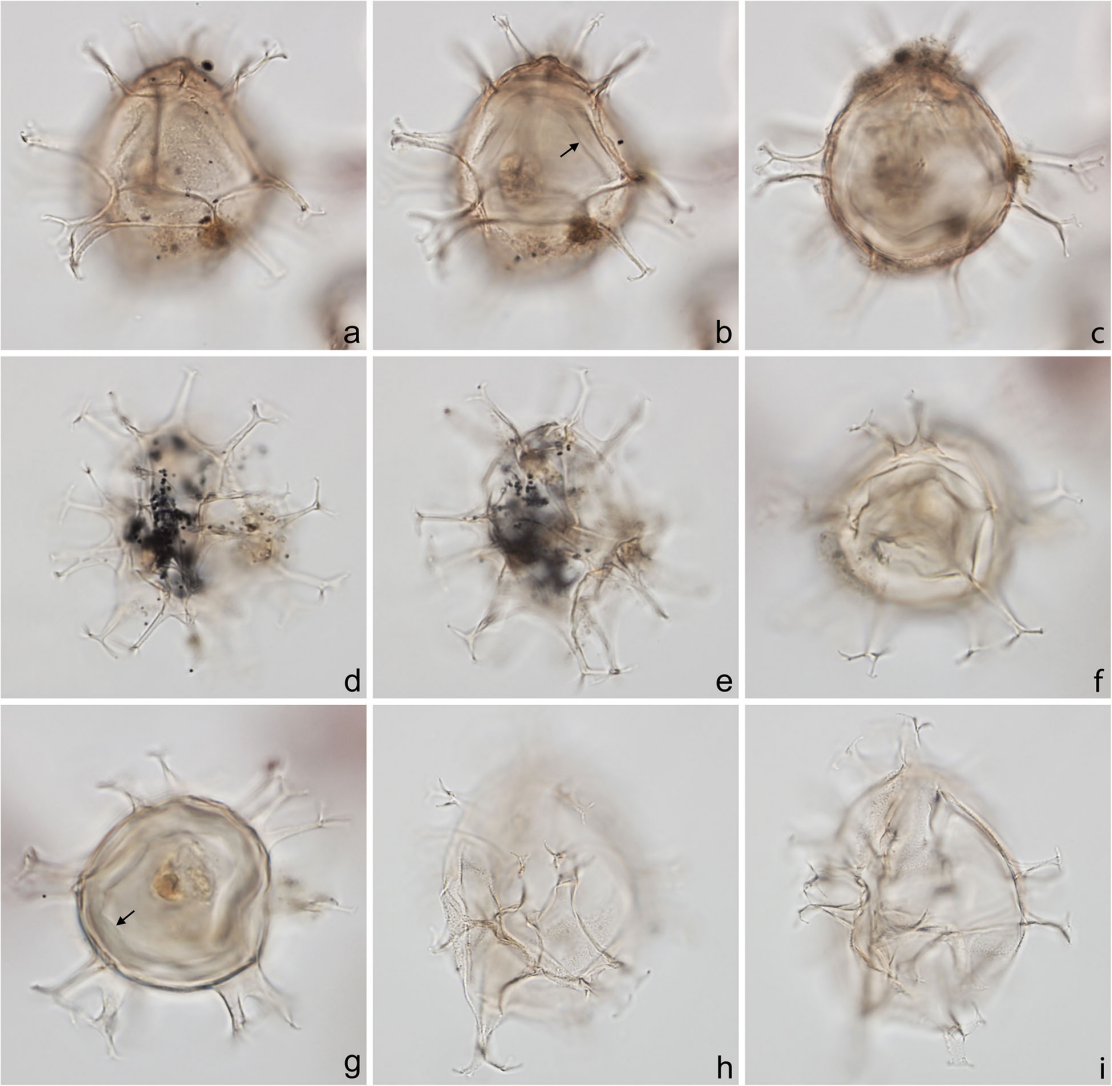
Dinoflagellate cysts from the Chiba composite section. Max. dia. = maximum diameter. EF = England Finder reference. **a**–**c** *Spiniferites pachydermus* sensu Mertens et al. (2015), dorsal view at upper (operculum in place), slightly lower and mid-focus; note apical protuberance and preserved endospore (arrow); central body length 43 μm, central body width 38 μm; sample TB2 00, slide 1 (10 μm) MJH, EF V16/4. **d**, **e** *Spiniferites ramosus* sensu Rochon et al. (1999), ventral-left-lateral view at upper and lower foci; central body length 33 μm; sample TB2 97, slide 3 (20 μm) MJH, EF M32/4. **f**, **g** *Spiniferites* sp. (a representative of *Spiniferites* spp. group A), apical view at upper and mid-foci, note preserved endospore (arrow); central body max. dia. 30 μm; sample TB2 97, slide 2 (10 μm) MJH, EF F13/0. **h**, **i** *Spiniferites* sp. B, view uncertain at upper and mid-foci; thin wall with distinctly granular periphragm surface; some processes tips petaloid; central body length 56 μm; sample TB2 97, slide 3 (20 μm) MJH, EF D34/0

**Fig. 8:**
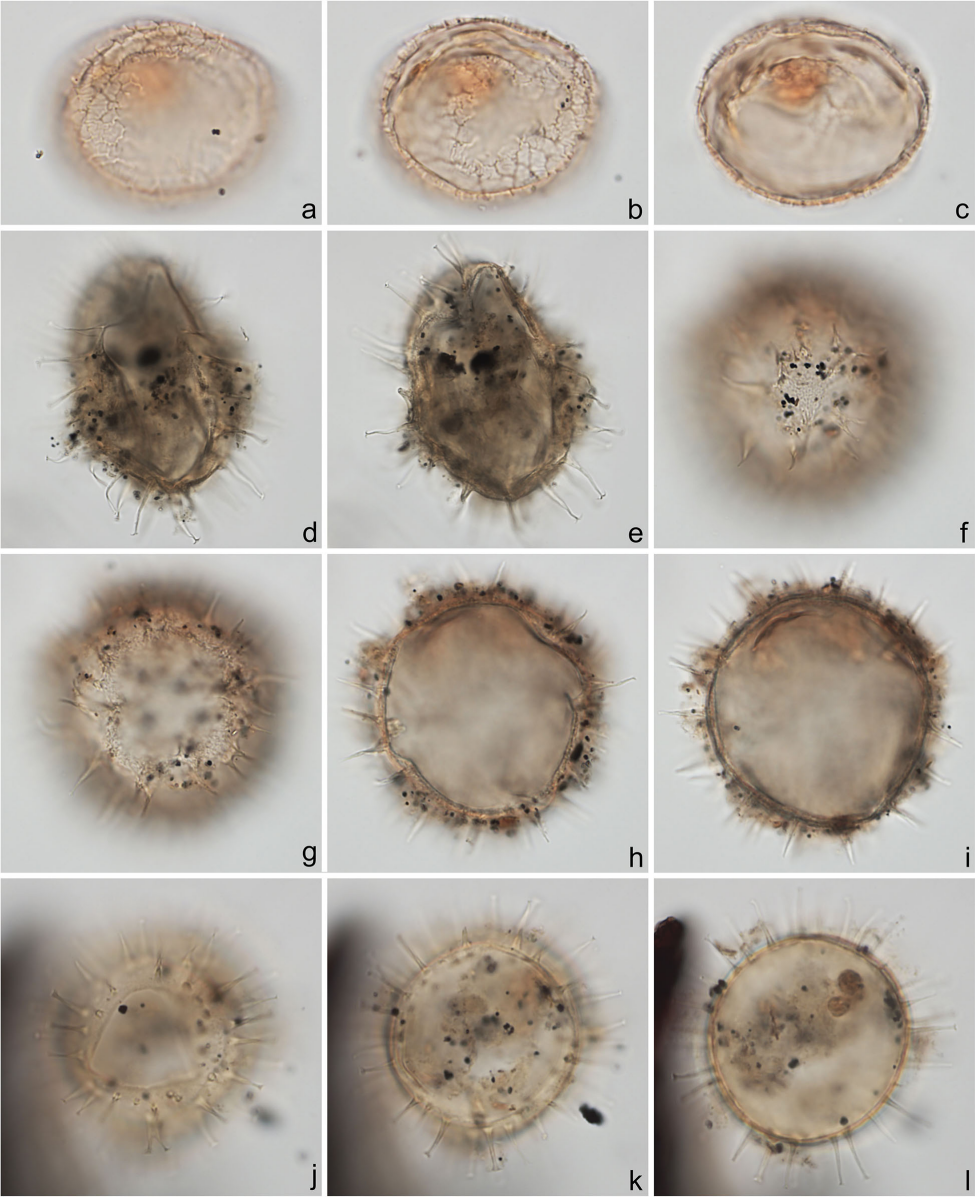
Dinoflagellate cysts from the Chiba composite section. Max. dia. = maximum diameter. EF = England Finder reference. **a**–**c** *Pyxidinopsis reticulata*, unknown view at upper through mid-foci; max. dia. (excluding ornament) 35 μm, max. crest height ~ 1.0 μm; sample TB 16, slide 1 (10 μm), EF S21/1. **d**, **e** *Operculodinium centrocarpum* sensu stricto, view uncertain, upper and mid-focus; processes have expanded fibrous bases and aculeate tips; central body wall ~ 1.0 μm thick with thick spongy-fibrous periphragm; central body length 59 μm; sample TB 78, slide 2 (10 μm), EF H20/1. **f**–**i** *Operculodinium israelianum*, apical? view; upper through mid-foci; note solid fibrous processes that may be acuminate or minutely expanded distally; central body max. dia. 45 μm; sample TB 88, slide 2 (10 μm), EF W37/4. **j**–**l** cyst of *Protoceratium reticulatum*, dorsal view at upper, slightly lower, and mid-foci; central body max. dia. 37 μm; sample TB2 61, slide 1 (10 μm), EF O18/3

**Fig. 9:**
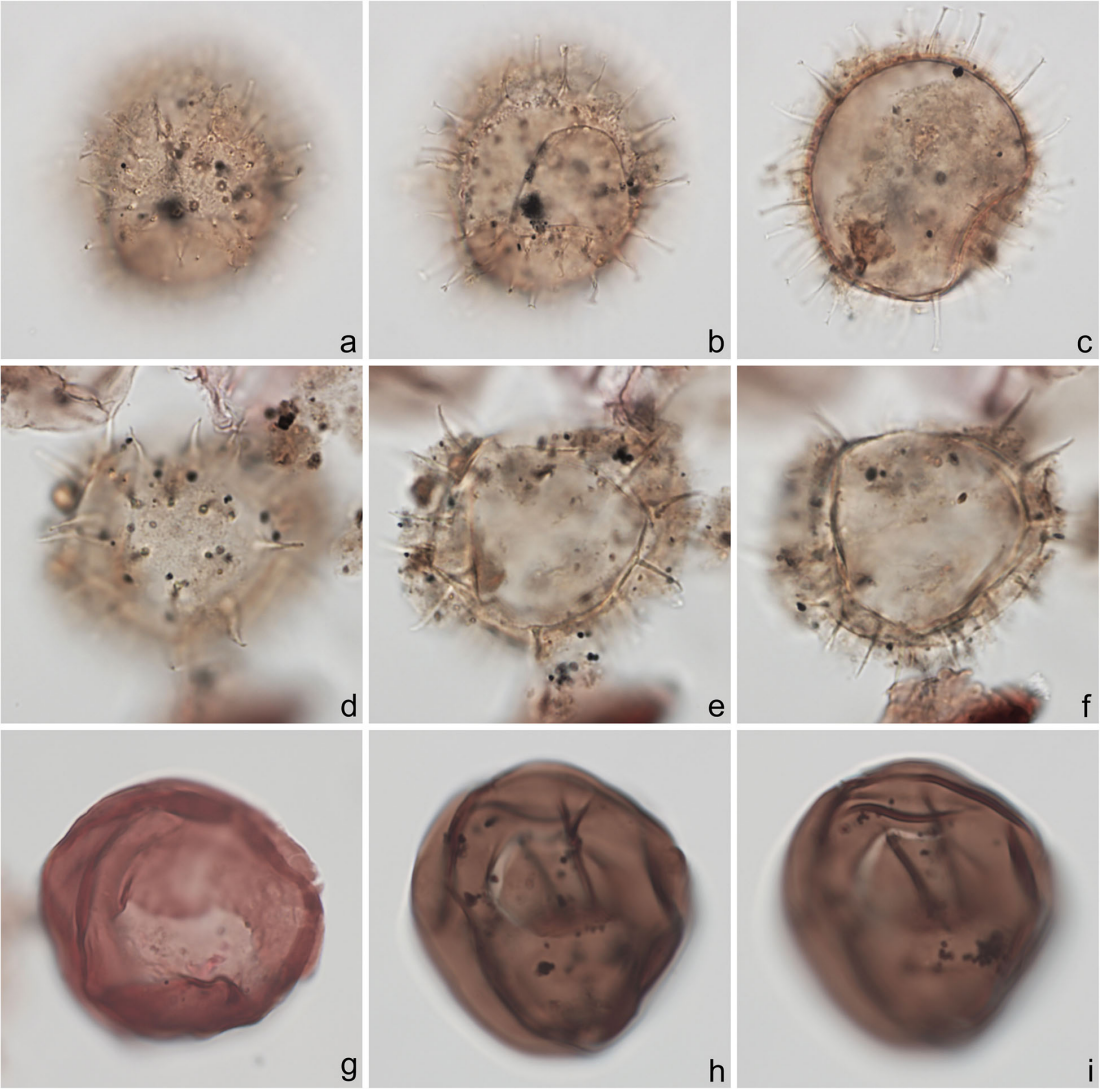
Dinoflagellate cysts from the Chiba composite section. Max. dia. = maximum diameter. EF = England Finder reference. **a**–**c** cyst of *Protoceratium reticulatum*, ventral view at upper, slightly lower, and mid-foci; **b** showing characteristic rounded adapical margin of detached operculum, also in ventral view, within cyst; central body max. dia. 40 μm; sample TB2 00, slide 3 (10 μm) MJH, EF Q9/4. **d**–**f** *Operculodinium*? *longispinigerum*, view uncertain; note finely granulate central body surface, solid tapering processes, somewhat flexuous with finely granulate surfaces distally and with circular bases; central body max. dia. 33 μm; sample TB 58, slide 1 (10 μm), EF J10/1. **g** *Brigantedinium cariacoense*, ventral view at mid-focus on archeopyle; cyst max. dia. 38 μm; sample TB 34, slide 1 (10 μm), EF H40/0. **h**, **i** *Brigantedinium* sp., dorsal view at mid- and lower focus; cyst max. dia. 36 μm; sample TB 72, slide 1 (10 μm), EF K38/1

**Fig. 10:**
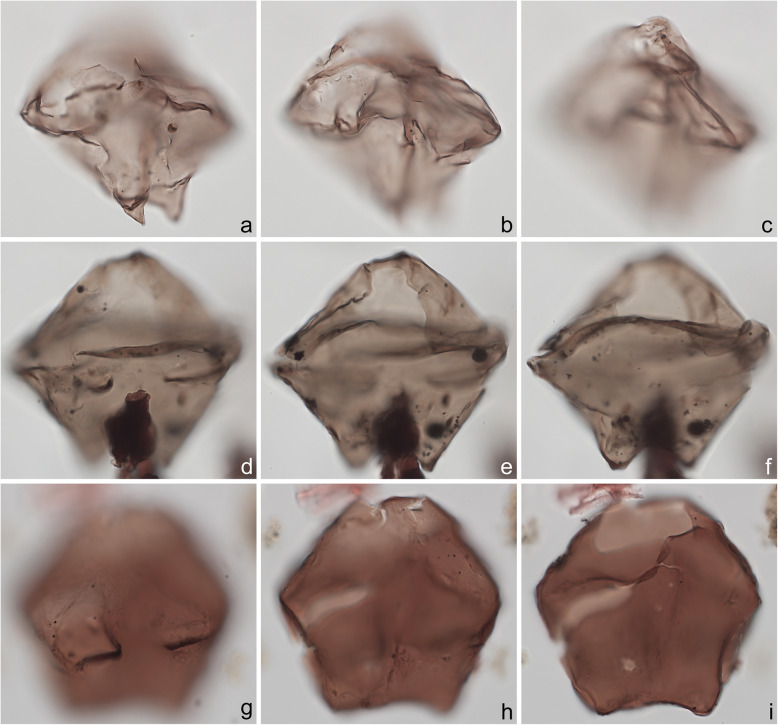
Dinoflagellate cysts from the Chiba composite section. Max. dia. = maximum diameter. EF = England Finder reference. **a**–**c** *Lejeunecysta* sp. A, dorsal view at upper, mid-, and lower foci; cyst length 67 μm; sample TB 50, slide 2 (10 μm), EF O40/3. **d**–**f** *Lejeunecysta* sp. B, ventral view at upper, mid-, and lower foci; cyst length 78 μm; sample TB2 59, slide 2 (10 μm), EF R37/1. **g**–**i** *Quinquecuspis concreta*, ventral view at upper through lower foci; cyst length 75 μm; sample TB 16, slide 2 (10 μm), EF N37/0

**Fig. 11:**
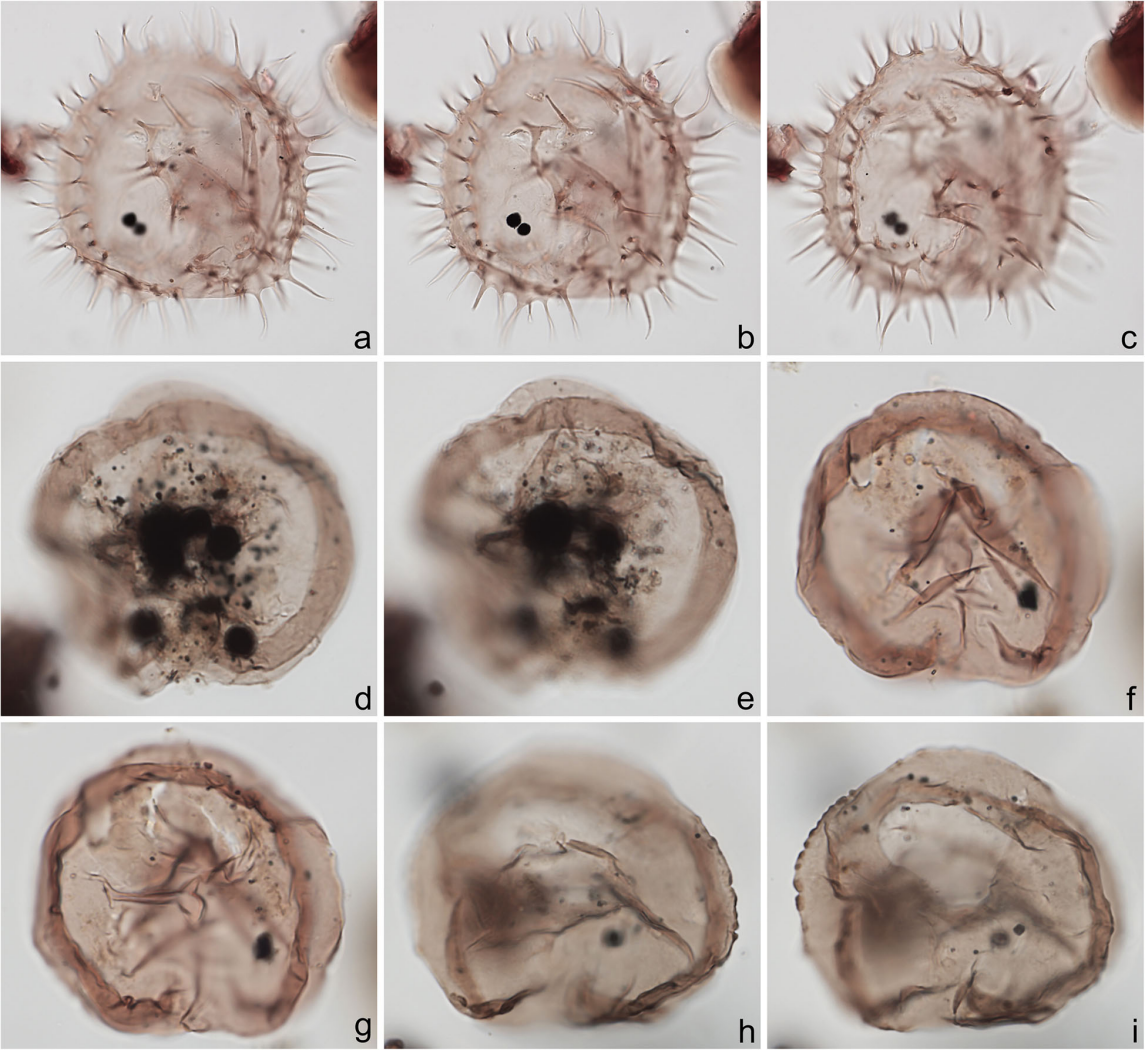
Dinoflagellate cysts from the Chiba composite section. Max. dia. = maximum diameter. EF = England Finder reference. **a**–**c** *Selenopemphix quanta*, antapical view at upper, mid-, and lower foci; central body width 75 μm, height 76 μm; sample TB2 97, slide 3 (20 μm) MJH, EF N13/3. **d**, **e** *Selenopemphix nephroides*, polar view at mid- and lower foci; cyst max. dia. 58 μm; sample TB 66, slide 2 (10 μm), EF G36/3. **f**–**i** *Selenopemphix undulata*; **f**, **g** antapical view at upper and lower foci showing weak cingular crest undulations; cyst width 49 μm; sample TB 12, slide 1 (20 μm), EF L29/3; **h**, **i** antapical view at upper and lower foci showing pronounced cingular crest undulations; cyst max. dia. 56 μm; sample TB 114, slide 1 (10 μm), EF O33/1

**Fig. 12:**
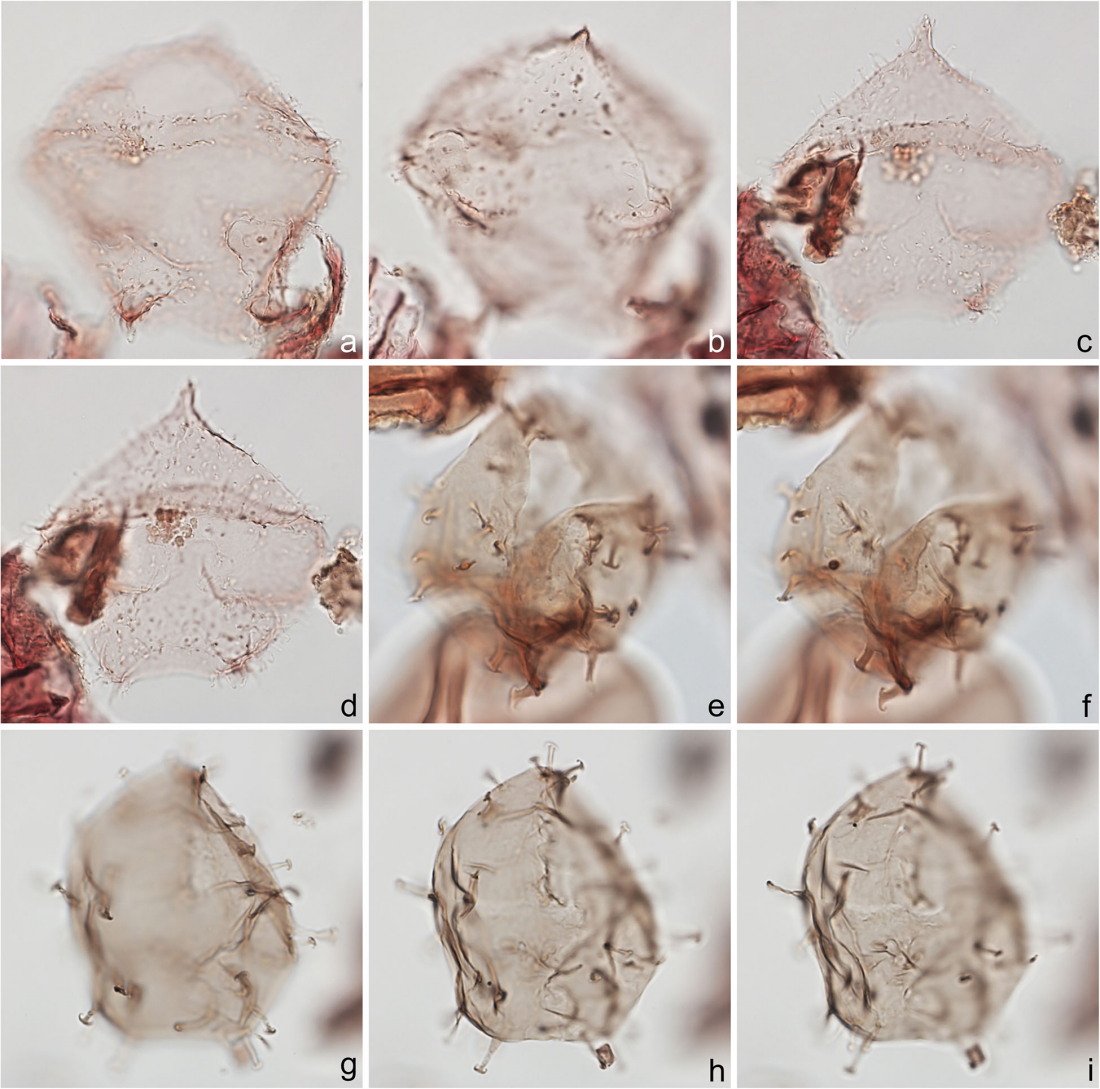
Dinoflagellate cysts from the Chiba composite section. Max. dia. = maximum diameter. EF = England Finder reference. **a**–**d** *Trinovantedinium applanatum*; **a**, **b** dorsal view at upper and lower foci; central body max. length 71 μm, width 72 μm; sample TB2 97, slide 2 (10 μm) MJH, EF L28/0; **c**, **d** dorsal view at upper and mid-foci; central body max. length 72 μm, width 67 μm; sample TB2 97, slide 2 (10 μm) MJH, EF Q24/2. **e**–**i** *Trinovantedinium* cf. *harpagonium* showing flattened bifurcate process terminations with strongly recurved tips; **e**, **f** ventral view at mid- and slightly lower focus; central body length 57 μm, width 50 μm; sample TB2 97, slide 4 (20 μm) MJH, EF G15/0; **g**–**i** ventral view at upper through lower foci; central body length 49 μm; sample TB 92, slide 1 (10 μm), EF W43/1

**Fig. 13:**
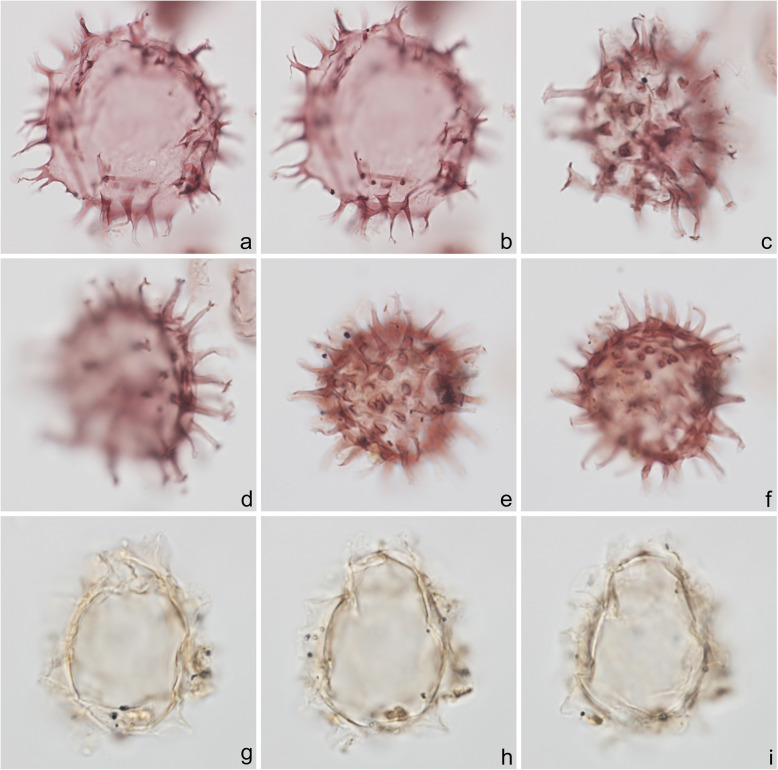
Dinoflagellate cysts from the Chiba composite section. Max. dia. = maximum diameter. EF = England Finder reference. **a**, **b** *Xandarodinium xanthum*, uncertain view at upper and lower foci; central body max. dia. 52 μm; sample TB 18, slide 1 (10 μm), EF F22/2. **c**–**f** *Echinidinium aculeatum*; **c**, **d** uncertain view at upper and lower foci; central body max. dia. 27 μm; sample TB 42 slide 1 (10 μm), EF N29/2; **e**, **f** uncertain view at upper and lower foci; central body max. dia. 23 μm; sample TB 16, slide 2 (10 μm), EF E42/0. **g**–**i** Cyst of *Scrippsiella trifida*, lateral view at upper, mid-, and lower foci; central body length 29 μm; sample TB 56, slide 2 (10 μm), EF R39/3

**Fig. 14:**
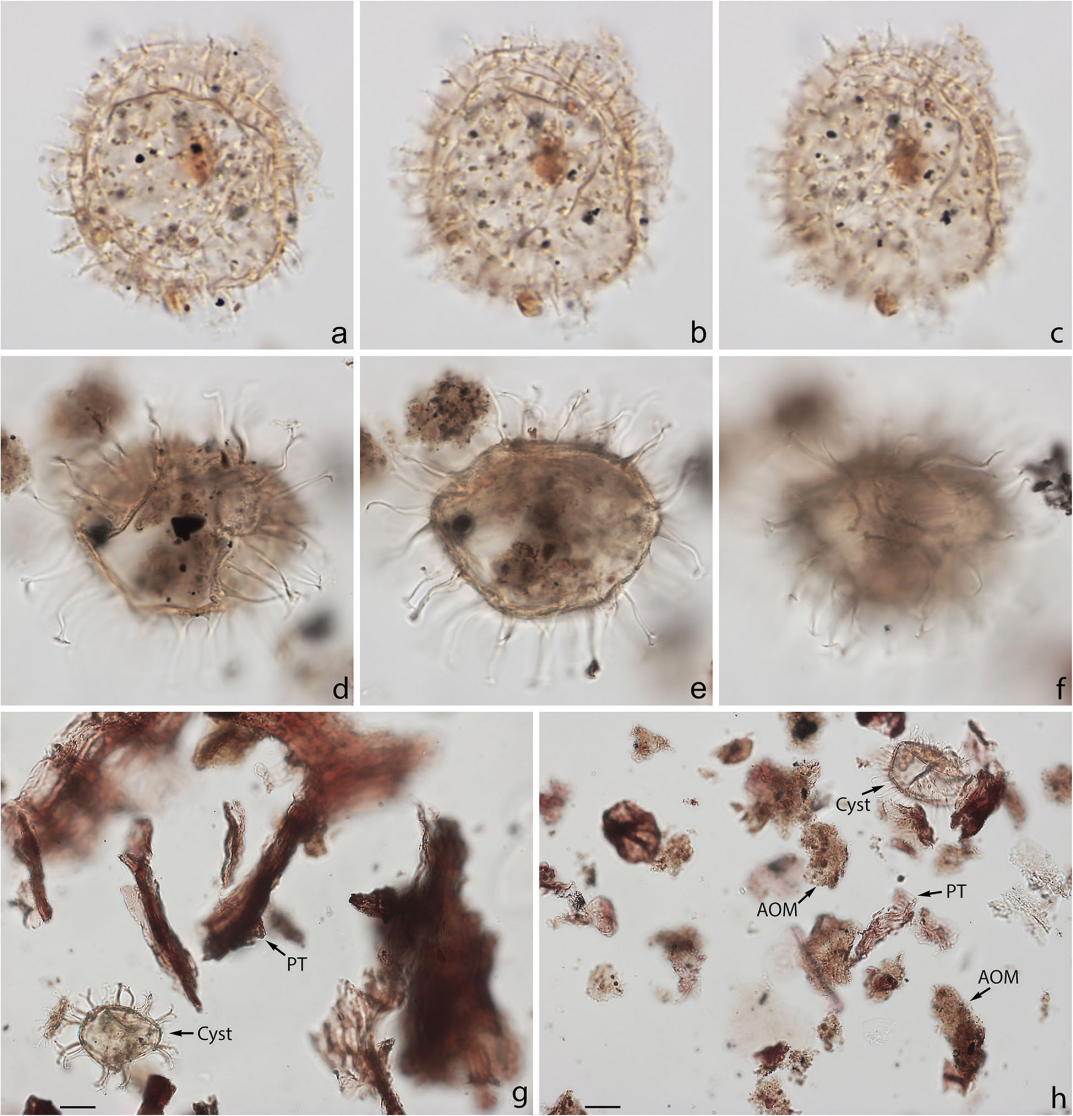
An acritarch (**a**–**c**), a presumed reworked dinoflagellate cyst (**d**–**f**), and palynofacies (**g**, **h**) from the Chiba composite section. Max. dia. = maximum diameter. EF = England Finder reference. **a**–**c** *Nannobarbophora walldalei*, uncertain view at upper, mid-, and lower foci; central body max. dia. 29 μm; sample TB2 30, slide 2 (10 μm), EF E15/3. **d**–**f** *Cleistosphaeridium placacanthum* (presumed reworked), apical view at upper, mid- and lower foci showing granulate central body surface with well-developed penitabular ridges; central body max. dia. 53 μm; sample TB 114, slide 1 (10 μm), EF P16/4. **g**, **h** Organic residue sieved at 10 μm showing (**g**) terrestrially dominated (sample TB2 105, slide 1, EF D21/4) and (**h**) AOM-dominated (sample TB 14, slide 1, EF D22/0) palynofacies; cyst = dinoflagellate cyst, AOM = amorphous organic matter, PT = plant tissues (woody tissues and cuticle); scale bar = 20 μm

### Taxonomy

The present study largely follows the taxonomy of Van Nieuwenhove et al. (2020), Mertens et al. (2020), and Limoges et al. (2020) for the dinoflagellate cysts, except that the cosmopolitan cyst morphotype (Fig. 8j–l, Fig. 9a–c) known in the Quaternary literature as *Operculodinium centrocarpum* sensu Wall and Dale (1966) is given its non-fossil name *Protoceratium reticulatum* following Paez-Reyes and Head (2013). It should not be confused with the larger and more robust *Operculodinium centrocarpum* sensu stricto (Fig. 8d, e), originally described from the Miocene of Australia (Deflandre and Cookson 1955). *Selenopemphix undulata* (Fig. 11f–i) was not always easy to separate from *Selenopemphix nephroides* (Fig. 11d, e) owing to the weakly expressed undulations on the cingular crests of some specimens. They were grouped as *Selenopemphix nephroides* + *undulata* in the counts. Owing to variable preservation, all smooth-walled round brown acavate cysts were labeled as “round brown cysts.” They are assumed to belong overwhelmingly to the genus *Brigantedinium* but because of folding, an archeopyle was seldom clearly observed and we cannot exclude the possibility that diplopsalid species bearing theropylic archeopyles (e.g., see Mertens et al. 2020) are also represented. On rare occasion where the archeopyle could be seen clearly, *Brigantedinium simplex* and *Brigantedinium cariacoense* (Fig. 9g) were both identified. Specimens of *Spiniferites mirabilis* (Fig. 5g–i) were identified as such regardless of whether the characteristic antapical flange was seen or not, as this feature is not always visible in polar orientations. *Spiniferites pachydermus* sensu Mertens et al. (2015) described from modern sediments of İzmir Bay, Turkey has a distinctive central body surface layer that is densely perforated, identical with that seen in the Chiba specimens (Fig. 6a–i, Fig. 7a–c). *Spiniferites pachydermus* described by Rossignol (1964) from the Pleistocene or Holocene of the Ashdod borehole, coastal plain, Israel, was described as having a thick central body wall (Rossignol 1964) but details of its structure and surface are not known, as discussed in Mertens et al. (2018). The Chiba specimens are considered not to belong to *Spiniferites pachydermu*s sensu stricto. *Spiniferites* spp. group A refers to several species with a thin central body wall and a smooth or weakly ornamented surface, and include *Spiniferites* cf. *belerius* (Fig. 5d–f) and *Spiniferites bulloides.* Owing to crumpling and unfavorable orientation, these specimens were grouped during counting as *Spiniferites* spp. group A. All other *Achomosphaera* and *Spiniferites* species are grouped as *Achomosphaera* + *Spiniferites* spp. indeterminate.

The taxonomy of the marine acritarch *Nanobarbophora walldalei* (Fig. 14a–c) follows Head (1996b, 2003).

### Statistical analyses

The concentrations (specimens per gram dry weight) of palynomorphs (Fig. 16) were estimated using the method of Stockmarr (1971), and the taxonomic richness and evenness of samples were also calculated (Fig. 16).

The taxonomic richness of a sample is the total number of taxa recorded in that sample. In the present study, this represents the number of dinoflagellate cyst taxa recorded in the count (usually ~ 300 specimens) for each sample.

Taxonomic evenness quantifies how equal taxa are in a sample, and greatest evenness is where all taxa in a sample share the same abundance. This study uses the Simpson *D* index which is not sensitive to taxonomic richness and is one of the most meaningful and robust diversity measures available (Magurran 2004). It is usually expressed as 1/*D* and ranges from 0 to 1 where higher values indicate greater evenness. To calculate taxonomic evenness, it is necessary first to derive Simpson’s *D* = ∑ *p*_*i*_^2^ where *p*_*i*_ is the proportion of each taxon in a sample. Then, Simpson’s measure of evenness (*E*_1/*D*_) is calculated by dividing the reciprocal form of *D* by the number of taxa in the sample:

*E*_1/*D*_ = (1/*D*) / *S*

where *S* = total number of taxa present in the sample (Magurran 2004).

## Results

**Fig. 15:**
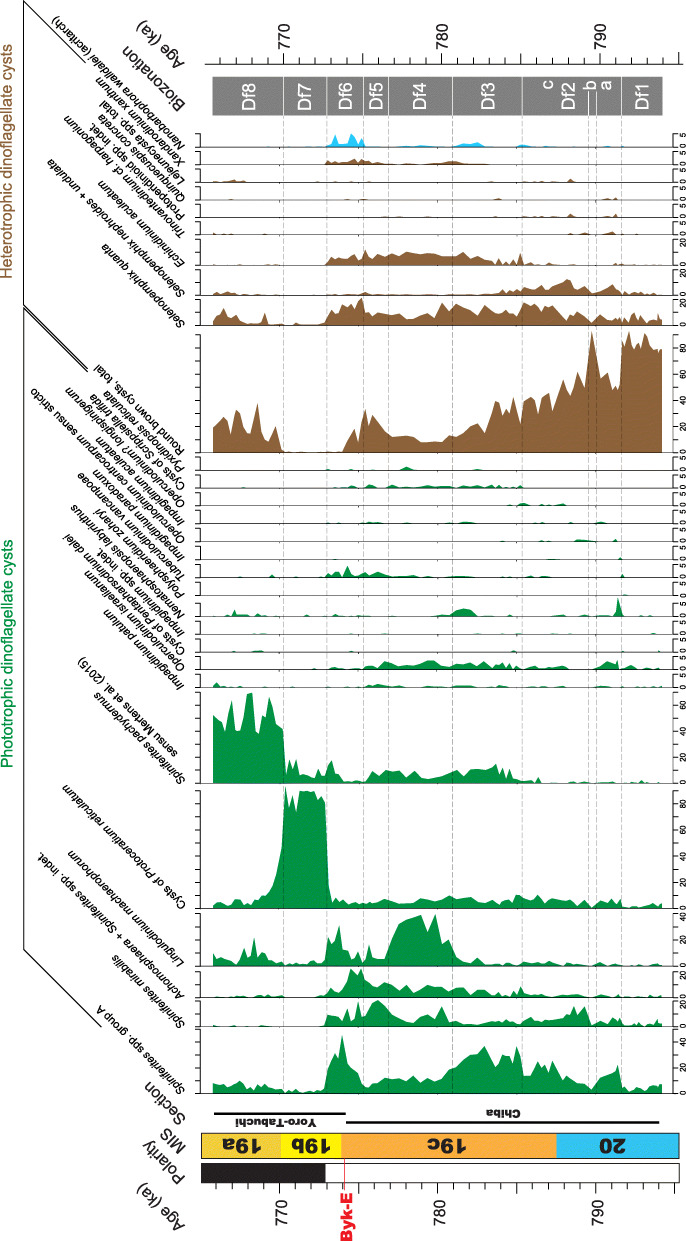
Relative abundance of dinoflagellate cysts and an acritarch from the Chiba and Yoro-Tabuchi sections plotted against the age model of Suganuma et al. (2018, in press). MIS and substage boundary positions are from Haneda et al. (2020b). All taxa are considered to be in situ. The position of Byk-E tephra bed is shown. The local dinoflagellate cyst biozonation (biozones Df1–8) is from the present study. All abundances are expressed as percentages of the total in-situ dinoflagellate cyst counts (or dinoflagellate cyst + *N*. *walldalei* counts for the abundances of *N. walldalei*). See also Fig. 16

**Fig. 16:**
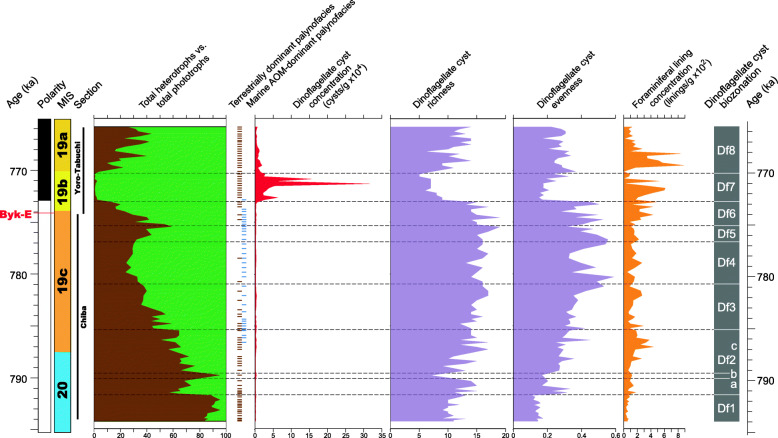
Marine palynology and palynofacies of the Chiba and Yoro-Tabuchi sections plotted against the age model of Suganuma et al. (2018, in press). MIS and substage boundary positions are from Haneda et al. (2020b). The position of Byk-E tephra bed is shown. The biozonation (biozones Df1–8) is from the present study, with total heterotrophs (brown) versus total phototrophs expressing the overall dinoflagellate cyst assemblage compositions. Dominant palynofacies component (brown line = terrestrial represented mostly by woody and cuticular plant tissues, blue line = marine represented by amorphous organic matter [AOM]) is given for each sample based on qualitative visual estimate. In a few samples, marine and terrestrial components co-dominate. Concentrations of dinoflagellate cysts (× 10,000) and benthic microforaminiferal linings (× 100) are per gram dry weight of sediment

All 107 samples were found to contain dinoflagellate cysts. A total of 29 in-situ dinoflagellate cyst taxa were counted along with the acritarch *Nannobarbophora walldalei*. The full names of all taxa reported and their systematic classification are listed in Table 1. Taxa are illustrated in Figs. 4, 5, 6, 7, 8, 9, 10, 11, 12, 13, 14. The relative abundances of all counted taxa are plotted in Fig. 15. Dinoflagellate cyst concentrations per gram dry weight, taxonomic richness and evenness of dinoflagellate cyst assemblages, and the concentrations per gram dry weight of benthic microforaminiferal linings are all given in Fig. 16. All raw counts and calculated values are given in Additional file 1.

**Table 1:** Dinoflagellate cyst and acritarch taxa recorded in the present study, and their classification and authorial attributions. All taxa are considered in situ with the exception of the genus *Cleistosphaeridium* which is presumed reworked

Subclass PERIDINIPHYCIDAE Fensome et al., 1993 Order GONYAULACALES Taylor, 1980 Suborder GONIODOMINEAE Fensome et al., 1993 Family PYROPHACACEAE Lindemann, 1928 Subfamily HELGOLANDINIOIDEAE Fensome et al., 1993	
Genus *Tuberculodinium* Wall, 1967 *Tuberculodinium vancampoae* (Rossignol, 1962) Wall, 1967. Fig. 4a.	
Subfamily PYRODINIOIDEAE Fensome et al., 1993	
Genus *Polysphaeridium* Davey and Williams, 1966 emend. Bujak et al., 1980 *Polysphaeridium zoharyi* (Rossignol, 1962) Bujak et al., 1980. Fig. 4b.	
Suborder GONYAULACINEAE (Autonym) Family GONYAULACACEAE Lindemann, 1928 Subfamily GONYAULACOIDEAE (Autonym)	
Genus A*chomosphaera* Evitt, 1963 *Achomosphera* spp. indet. Grouped with *Achomosphera*+*Spiniferites* spp. indet. in the counts.	
Genus *Impagidinium* Stover and Evitt, 1978 *Impagidinium aculeatum* (Wall, 1967) Lentin and Williams, 1981. Fig. 4e, f. *Impagidinium paradoxum* (Wall, 1967) Stover and Evitt, 1978. Fig. 4g–i. *Impagidinium patulum* (Wall, 1967) Stover and Evitt, 1978. Fig. 4j–l.	
Genus *Nematosphaeropsis* Deflandre and Cookson 1955 emend. Wrenn, 1988 *Nematosphaeropsis labyrinthus* (Ostenfeld, 1903) Reid, 1974. Fig. 5a–c.	
Genus *Spiniferites* Mantell, 1850 emend. Sarjeant, 1970 *Spiniferites* sp. cf. *S. belerius* Reid, 1974. Fig. 5d–f. Grouped with *Spiniferites* spp. group A in the counts. *Spiniferites mirabilis* (Rossignol 1964) Sarjeant, 1970 emend. Limoges et al., 2018. Fig. 5g–i. *Spiniferites pachydermus* sensu Mertens et al. (2015). Non *Spiniferites pachydermus* (Rossignol 1964) Reid, 1974. Figs. 6a–i, 7a–c. *Spiniferites ramosus* (Ehrenberg, 1837) Mantell, 1854 sensu Rochon et al. 1999. Fig. 7d, e. Grouped with *Spiniferites* spp. group A in the counts. *Spiniferites* sp. Fig. 7f, g. Grouped with *Spiniferites* spp. group A in the counts. *Spiniferites* spp. group A. *Spiniferites* sp. B. Fig. 7h, i. Not recorded in the counts. *Spiniferites* spp. indet. Grouped with *Achomosphera*+*Spiniferites* spp. indet. in the counts.	
Family LINGULODINIACEAE Sarjeant and Downie, 1974 emend. Gu et al. in Zhang et al., 2020	
Genus *Lingulodinium* Wall, 1967 emend. Dodge, 1989 *Lingulodinium machaerophorum* (Deflandre and Cookson 1955) Wall, 1967. Fig. 4c, d.	
Genus *Pyxidinopsis* Habib, 1976 *Pyxidinopsis reticulata* McMinn and Sun, 1994 emend. Marret and de Vernal, 1997. Fig. 8a–c.	
Family PROTOCERATIACEAE Lindemann 1928 emend. Gu and Mertens, 2020	
Genus *Operculodinium* Wall, 1967 emend. Matsuoka et al., 1997 *Operculodinium centrocarpum* (Deflandre and Cookson 1955) Wall, 1967 sensu stricto. Fig. 8d, e. *Operculodinium israelianum* (Rossignol, 1962) Wall, 1967. Fig. 8f–i. *Operculodinium*? *longispinigerum* Matsuoka, 1983. Fig. 9d–f.	
Genus *Protoceratium* Bergh, 1881 emend. Gu and Mertens, 2020 *Protoceratium reticulatum* (Claparède and Lachmann, 1859) Bütschli, 1885. Figs. 8j–l, 9a–c. *Protoceratium reticulatum* – short processes. This morphotype occurs only rarely and was grouped with *Protoceratium reticulatum* in the counts.	
Family UNCERTAIN	
Genus *Cleistosphaeridium* Davey et al., 1966 emend. Eaton et al. 2001 *Cleistosphaeridium placacanthum* (Deflandre and Cookson 1955) Eaton et al. 2001. Fig. 14d–f. Presumed reworked. Grouped with *Cleistosphaeridium* spp. in the counts. *Cleistosphaeridium* spp. Presumed reworked.	
Order PERIDINIALES Haeckel, 1894 Suborder PERIDINIINEAE (Autonym) Family PROTOPERIDINIACEAE Balech, 1988 Subfamily PROTOPERIDINIOIDEAE Balech, 1988	
Genus *Brigantedinium* Reid, 1977 ex Lentin and Williams, 1993. Grouped with round brown cysts in the counts. *Brigantedinium cariacoense* (Wall, 1967) Lentin and Williams, 1993. Fig. 9g. Grouped with round brown cysts in the counts. *Brigantedinium simplex* Wall, 1965 ex Lentin and Williams, 1993. Grouped with round brown cysts in the counts. *Brigantedinium* sp. Fig. 9h, i. Grouped with round brown cysts in the counts.	
Genus *Lejeunecysta* Artzner and Dörhöfer, 1978, emend. Lentin and Williams, 1976 *Lejeunecysta* sp. A. Fig. 10a–c. Grouped with *Lejeunecysta* spp. total in the counts. *Lejeunecysta* sp. B. Fig. 10d–f. Grouped with *Lejeunecysta* spp. total in the counts.	
Genus *Quinquecuspis* Harland, 1977 *Quinquecuspis concreta* (Reid, 1977) Harland, 1977. Fig. 10g–i.	
Genus *Selenopemphix* Benedek, 1972 emend. Head, 1993, nom. cons. *Selenopemphix quanta* (Bradford, 1975) Matsuoka 1985. Fig. 11a–c. *Selenopemphix nephroides* Benedek, 1972 emend. Bujak in Bujak et al., 1980. Fig. 11d, e. Grouped with *Selenopemphix nephroides* + *undulata* in the counts. *Selenopemphix undulata* Verleye et al., 2011. Fig. 11f–i. Grouped with *Selenopemphix nephroides* + *undulata* in the counts.	
Genus *Trinovantedinium* Reid, 1977, emend. de Verteuil and Norris, 1992 *Trinovantedinium applanatum* (Bradford, 1977) Bujak and Davies, 1983. Fig. 12a–d. Not seen during counting. *Trinovantedinium* sp. cf. *T*. *harpagonium* de Verteuil and Norris, 1992. Fig. 12e–i.	
Genus *Xandarodinium* Reid, 1977 *Xandarodinium xanthum* Reid, 1977. Fig. 13a, b.	
Subfamily UNCERTAIN	
Genus *Echinidinium* Zonneveld, 1997 ex Head et al., 2001 *Echinidinium aculeatum* Zonneveld, 1997 ex Mertens et al. 2020. Fig. 13c–f.	
Family THORACOSPHAERACEAE Schiller, 1930 emend. Tangen in Tangen et al., 1982	
Genus *Pentapharsodinium* Indelicato and Loeblich III, 1986 emend. Montresor et al., 1993 *Pentapharsodinium dalei* Indelicato and Loeblich III, 1986	
Genus *Scrippsiella* Balech, 1959 ex Loeblich III, 1965 *Scrippsiella trifida* Lewis, 1991 ex Head, 1996. Fig. 13g–i.	
Group ACRITARCHA Evitt, 1963	
Genus *Nannobarbophora* Habib and Knapp, 1982 *Nannobarbophora walldalei* Head, 1996. Fig. 14a–c.

The preservation of dinoflagellate cysts varies from good to moderate, with specimens often affected by some folding/compression and breakage, and the heterotrophs sometimes showing partial corrosion. Some specimens have preserved endosperms (Fig. 7b, g). Cyst concentrations vary from 907 to 317,932 cysts per gram dry weight. Opaque mineral inclusions, presumably diagenetic pyrite, were found in all samples. The palynofacies are characterized throughout by common to dominant terrestrial components, mostly woody and cuticular tissues but also spores and pollen (Fig. 14g). Marine AOM (Fig. 14h) dominates or co-dominates much of the interval from sample TB 63 (786.60 ka) to TB2 24 (772.85 ka). Dinoflagellate cysts are a relatively minor component of the palynofacies, and counting them is hence very time consuming for most samples.

Few reworked dinoflagellate cysts were recognized; the most distinctive being specimens of the genus *Cleistosphaeridium* (Fig. 14d–f) which occur as isolated specimens (Additional file 1). These specimens have similar preservation to the in-situ cysts, and are considered reworked primarily because *Cleistosphaeridium* is a predominantly Eocene–Miocene genus (Eaton et al. 2001).

All assemblages are dominated by neritic taxa, with specimens of the oceanic genus *Impagidinium* (Fig. 4e–l) and oceanic *Nematosphaeropsis labyrinthus* (Fig. 5a–c) occurring sporadically and usually rarely. Based on a qualitative assessment of the in-situ dinoflagellate cyst assemblages, cyst concentrations, and abundance of the acritarch *Nanobarbophora walldalei*, eight informal assemblage biozones (Fig. 11) are described in ascending stratigraphic order, as given below, with sample depths expressed as meters below or above the Byk-E tephra bed and astronomical ages based on Suganuma et al. (2018, in press). Qualitative observations on the palynofacies are given for each (sub)biozone, and the dominant palynofacies component for each sample is shown on Figs. 16, 18c, and 19h.

### Dinoflagellate cyst assemblage biozone 1 (Df1)

Samples: TB 145–92; depth − 14.5 to − 9.18 m (− 12.25 to − 9.18 m without sand [turbidite] layers); age 794.21–791.72 ka; upper MIS 20.

Description: Heterotrophs in general, and round brown cysts (76–93%) in particular, dominate assemblages and have their highest sustained abundance (up to 95%) in this biozone. The heterotroph *Selenopemphix quanta* (1.3–9.7%) is the second most abundant species, followed by *Selenopemphix nephroides* + *undulata* (0–3.1%). Phototrophic species are rare throughout, and include *Spiniferites* spp. group A (1.3–7.4%). Cyst concentrations fluctuate from 907 to 2464 cysts/g. All samples are dominated by plant tissues and terrestrial sporomorphs, with marine AOM occurring in trace to rare quantities in nearly all samples.

### Dinoflagellate cyst assemblage biozone 2 (Df2)

Samples: TB 90–59; depth − 8.98 to − 5.85 m; age 791.55–785.47 ka; upper MIS 20–lower MIS 19c.

Description: This biozone spans Termination IX and is mainly characterized by a progressive decline in the relative abundance of round brown cysts from 92 to 29% and an overall rise in *Selenopemphix quanta* from 1.7 to 18%. Biozone 2 is subdivided into sub-biozones Df2a, Df2b, and Df2c.

### Sub-biozone Df2a

Samples: TB 90–80; depth − 8.98 to − 8.00 m; age 791.55–790.32 ka; upper MIS 20.

Description: This subzone begins with a sharp drop and then rise in round brown cysts (48–61%) and a corresponding rise and then drop in *Spiniferites* spp. group A (12–22%). There is a slight increase also in *Spiniferites mirabilis* (2.1–7.3%), the cysts of *Protoceratium reticulatum* (3.1–7.3%), and *Operculodinium israelianum* (0–3.5%). *Nematosphaeropsis labyrinthus* rises to 7.3% near the base of this subzone. In-situ cyst concentrations fluctuate from 1024 to 1825 cysts/g. All samples are dominated by plant tissues and terrestrial sporomorphs, with marine AOM occurring in rare to moderate quantities in most samples.

### Sub-biozone Df2b

Sample: TB 78; depth − 7.75 m; age 789.76 ka; upper MIS 20.

Description: This subzone is represented by a single sample showing a distinct rise in round brown cysts (92%) and corresponding drop in all other taxa. The cyst concentration is 3670 cysts/g. The sample is dominated by plant tissues and terrestrial sporomorphs, with rare marine AOM.

### Sub-biozone Df2c

Samples: TB 76–59; depth − 7.55 to − 5.85 m; age 789.31–785.47 ka; upper MIS 20–lower MIS 19c.

Description: This subzone records declining abundances of round brown cysts from 61 to 29%, and increasing abundances of *Selenopemphix quanta* to 18%. *Selenopemphix nephroides* + *undulata* has its highest abundance (12%) in this biozone. Among the phototrophs, *Spiniferites* spp. group A increases from 7.6 to 25%, and *Spiniferites mirabilis* increases to 16% at the base of the subzone but declines to 4.6% toward the top. Cysts of *Protoceratium reticulatum* resume low abundances of 1.6–10%. Cyst concentrations fluctuate from 1125 to 3682 cysts/g. Samples from the base of the subzone to sample TB 70 (787.96 ka) are dominated by plant tissues and terrestrial sporomorphs, with marine AOM occurring mostly in rare to moderate quantities. From sample TB 66 (787.30 ka) to the top of the subzone, marine AOM becomes more common, and is mostly abundant to dominant from sample TB 63 (786.60 ka) to the top of the subzone.

### Dinoflagellate cyst assemblage biozone 3 (Df3)

Samples: TB 58–40; depth − 5.75 to − 3.95 m; age 785.20–781.20 ka; MIS 19c.

Description: Declining abundances of heterotrophs, including round brown cysts from 42 to 12% and *Selenopemphix nephroides* + *undulata* from 4.7 to 0.2%, elevated abundances of the heterotroph *Echinidinium aculeatum* (0.3–10%), and high abundances of the phototroph *Spiniferites* spp. group A (20–37%), characterize this zone. Occurring throughout the zone are *Selenopemphix quanta* (6.3–17%), *Spiniferites pachydermus* sensu Mertens et al. (2015) (1.6–15%), *Operculodinium israelianum* (0–3.0%), and cysts of *Scripsiella trifida* (0–1.3%). *Xandarodinium xanthum* reaches 1.3% at the top of the zone. Cyst concentrations fluctuate from 916 to 4128 cysts/g. Plant tissues and terrestrial sporomorphs in general co-dominate with marine AOM throughout this zone.

### Dinoflagellate cyst assemblage biozone 4 (Df4)

Samples: TB 38–22; depth − 3.75 to − 2.15 m; age 780.74–777.13 ka; MIS 19c.

Description: The most notable feature of this zone is the sustained acme of *Lingulodinium machaerophorum* (17–39%) which is accompanied by a drop in the abundance of both *Spiniferites* spp. group A (to 8.3%) and round brown cysts (to 8.0%). The heterotroph *Echinidinium aculeatum* has sustained significant abundances (7.0–10%). The phototrophs *Spiniferites mirabilis* (2.9–16%), *Achomosphaera* + *Spiniferites* spp. indet. (3.4–10%), and *Operculodinium israelianum* (0.8–3.3%) all attain relatively high values in this zone, with the heterotroph *Selenopemphix quanta* showing a slight decline (to 4.6%). Cyst concentrations fluctuate from 1631 to 3233 cysts/g. Marine AOM dominates most samples through this zone, although plant tissues and terrestrial sporomorphs occur commonly in most samples.

### Dinoflagellate cyst assemblage biozone 5 (Df5)

Samples: TB 20–12; depth − 1.95 to − 1.15 m; age 776.68–775.44 ka; MIS 19c.

Description: This zone is characterized by an increase in *Spiniferites mirabilis* to peak values (21%) and a sharp decline in *Lingulodinium machaerophorum* (to 5.2%). *Spiniferites* spp. group A drops to 4.7%. *Spiniferites pachydermus* sensu Mertens et al. (2015) has values of 1.1–10%. Species of the oceanic genus *Impagidinium* in total rise to 1.8%, and *Tuberculodinium vancampoae* increases to between 0.7 and 2.3%. A rise in round brown cysts to 33% is also recorded, and *Echinidinium aculeatum* maintains abundances of 4.7–12%. Cyst concentrations fluctuate from 1838 to 2585 cysts/g. Marine AOM dominates all samples through this zone, with plant tissues and terrestrial sporomorphs occurring commonly in most samples.

### Dinoflagellate cyst assemblage biozone 6 (Df6)

Samples: TB 10–2, TB2 34–26; depth − 0.95–0.95 m; age 775.21–773.07 ka; MIS 19c–b.

Description: An increase in *Spiniferites* spp. group A to 45%, representing its acme in this succession, characterizes this zone, together with increased abundances of *Achomosphera* + *Spiniferites* spp. indet. to 22% in its lower part and *Lingulodinium machaerophorum* rising to 31% in the upper part of the zone. *Tuberculodinium vancampoae* has its highest values in this zone, reaching 4.4%. *Spiniferites mirabilis* maintains moderately high but fluctuating values of 4.1–20%. *Spiniferites pachydermus* sensu Mertens et al. (2015) rises to 17% near the top of this zone. Among the heterotrophs, round brown cysts decrease to 0.3% through this zone, whereas *Echinidinium aculeatum* has moderate values (2.6–8.4%) but is absent or rare above this zone. Both *Selenopemphix quanta* (up to 21%) and *Xandarodinium xanthum* (up to 2.3%) have their acmes in this zone, as does the acritarch *Nanobarbophora walldalei* (up to 5.0% of the total in-situ dinoflagellate cysts). Cyst concentrations fluctuate from 1969 to 4839 cysts/g. From the base up to sample TB2-32 (773.75 ka), marine AOM dominates with plant tissues and terrestrial sporomorphs being common to abundant. Above this, plant tissues and terrestrial sporomorphs dominate to the top of the zone, with marine AOM common to abundant.

### Dinoflagellate cyst assemblage biozone 7 (Df7)

Samples: TB2 24–02; depth 1.15–3.35 m; age 772.85–770.37 ka; MIS 19b.

Description: The sustained dominance of *Protoceratium reticulatum* cysts (73–93%) characterizes this zone. These cysts rise abruptly at the base of the biozone having not previously attained abundances higher than 17% (in the highest sample of the subjacent zone), and fall abruptly at the top of the biozone. The only other relatively persistent species in this zone are *Spiniferites* spp. group A (0.3–4%), *Lingulodinium machaerophorum* (0.3–4.7%), and *Spiniferites pachydermus* sensu Mertens et al. (2015) (3.7–18%), and the heterotrophs, round brown cysts (0.3–1.0%) and *Selenopemphix quanta* (0–2.0%). This biozone records by far the highest cyst concentrations, at 21,639–317,932 cysts/gram. The lowest sample (TB2-24) is dominated by marine AOM, with plant tissues and terrestrial sporomorphs being abundant. From sample TB2-22 (772.62 ka) to the top of the zone, samples are dominated by plant tissues and terrestrial sporomorphs, with marine AOM becoming rare from sample TB2-14 (771.72 ka) upwards.

### Dinoflagellate cyst assemblage biozone 8 (Df8)

Samples: TB2 00, TB2 39–109; depth 3.55–7.40 m; age 770.14–765.80 ka; uppermost MIS 19b, MIS 19a.

Description: A steep decline in the cysts of *Protoceratium reticulatum* from 93% top of biozone 7 to 47% at the base of biozone 8, and declining to a minimum of 2.0% in this biozone, and the rapid rise to dominance of *Spiniferites pachydermus* sensu Mertens et al. (2015) (38–69%) throughout this zone, are defining features. Other species with a notable presence are *Spiniferites* spp. group A (2.3–9.9%), *Lingulodinium machaerophorum* (1.0–22%), *Nematosphaeropsis labyrinthus* (0–2.6%), and the heterotrophic taxa, round brown cysts (2.3–38%), *Selenopemphix quanta* (0.3–13%), and *Selenopemphix nephroides + undulata* (up to 2.9%). Cyst concentrations fluctuate from 2459 to 19,205 cysts/g, representing a significant drop from concentrations in subjacent biozone Df7. Samples are dominated by plant tissues and terrestrial sporomorphs, with marine AOM occurring in rare to trace amounts.

## Discussion

All assemblages are dominated by neritic dinoflagellate cysts, with species of the oceanic genus *Impagidinium* and predominantly oceanic *Nematosphaeropsis labyrinthus* usually occurring rarely at most. The abundance of terrestrial material, mostly woody and cuticular tissues but also spores and pollen, indicates important downslope transport from the shelf. This taphonomic process likely accounts also for some of the neritic dinoflagellate cysts, but a strong primary paleoecological signal is clearly preserved. Marine AOM, which dominates or co-dominates the palynofacies for much of the interval from 786.60 ka to 772.85 ka, closely follows the total organic carbon/total nitrogen (C/N) and δ^13^C_org_ records and radiolarian concentrations (Fig. 19) and therefore seems to reflect elevated organic production.

The presence of opaque mineral inclusions, presumably diagenetic pyrite, in all samples reflects oxygen-depleted conditions below the water/sediment interface, perhaps facilitated by the rapid sedimentation rates, although the presence of benthic microforaminiferal linings in all but one sample (Fig. 16) and intense bioturbation throughout (Nishida et al. 2016) attest to oxygenated bottom waters and near-surface sediment. The pyrite presumably formed below the interval of intense bioturbation.

**Fig. 17:**
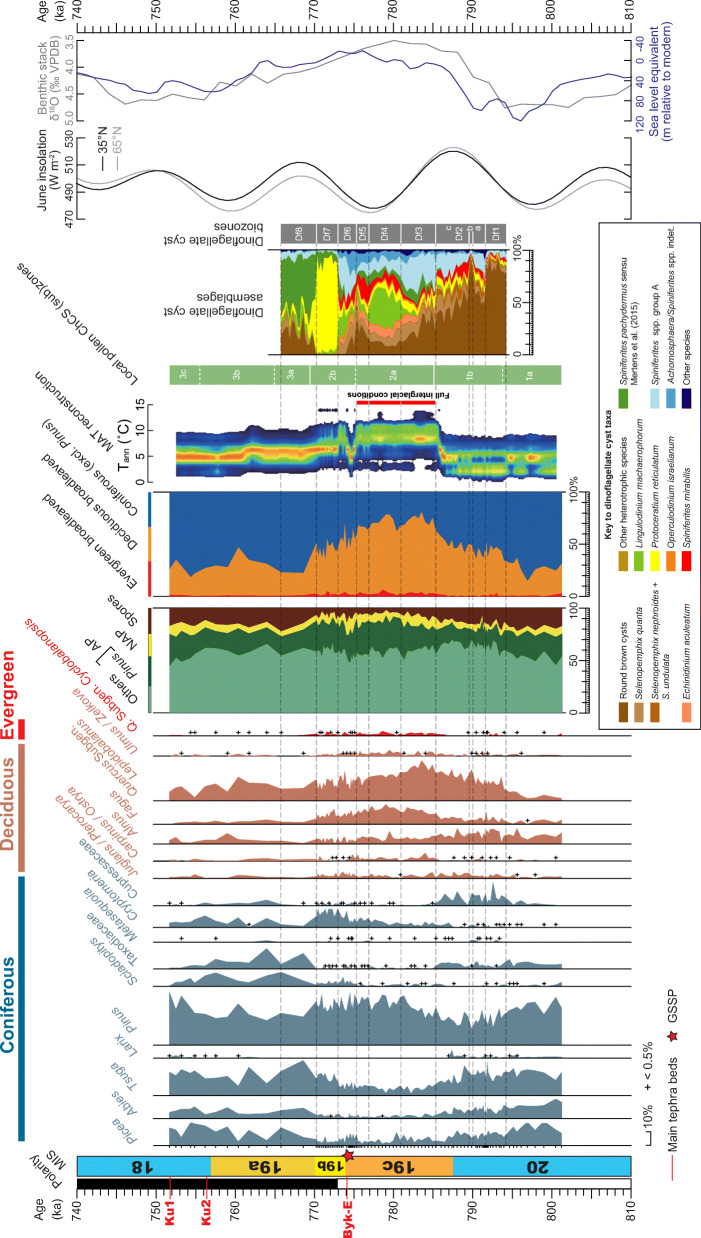
Dinoflagellate cyst synthetic diagram showing the most abundant taxa and local assemblage biozonation (this study) plotted alongside the major pollen taxa and quantified variations in the mean annual temperature (*T*_ann_) based on pollen and using the modern analogue technique (from fig. 7 of Suganuma et al. 2018). The age model is based on Suganuma et al. (2018) and MIS and substage boundary positions are from Haneda et al. (2020b). Included for comparison are the LR04 benthic stack (Lisiecki and Raymo 2005), the eustatic curve of Elderfield et al. (2012), and the 65°N and 35°N June insolation curves (from Laskar et al. 2004)

**Fig. 18:**
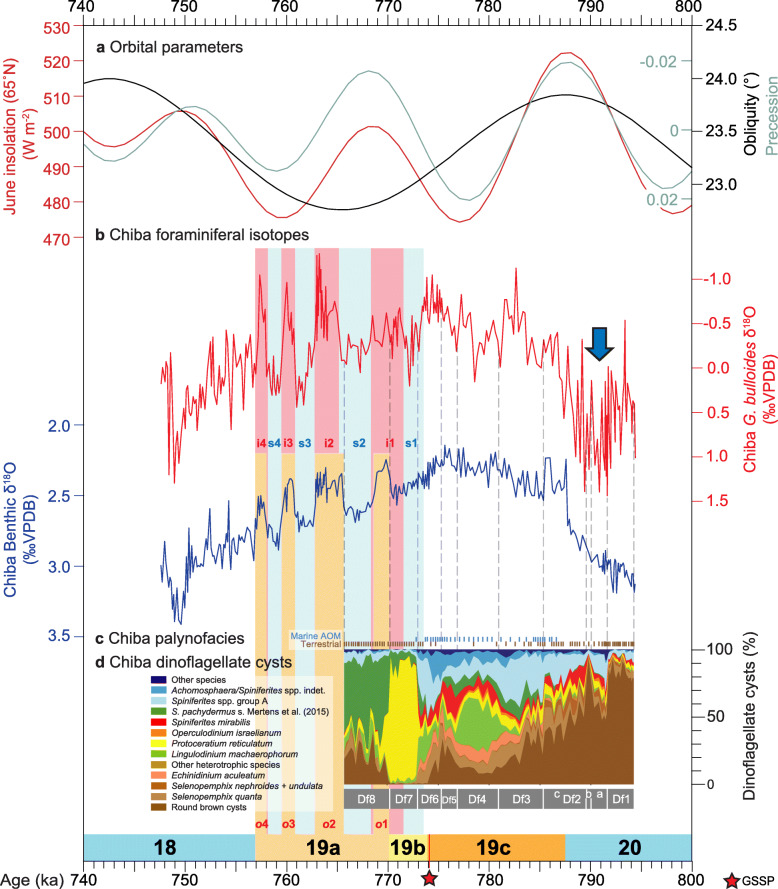
Dinoflagellate cyst synthetic diagram (this study) compared with the foraminiferal δ^18^O records of the Chiba composite section. **a** Insolation at 65°N in June, and precession and obliquity parameters (Laskar et al. 2004). **b** Planktonic (*G. bulloides*) and benthic foraminiferal δ^18^O records of the Chiba composite section (Haneda et al. 2020b) based on the age model of Suganuma et al. (2018). **c** Dominant palynofacies component (brown line, terrestrial; blue line, marine amorphous organic matter [AOM]) from Fig. 16. **d** Dinoflagellate cyst synthetic diagram showing the most abundant taxa and local assemblage biozonation (this study). Substage classification of MIS 19 with stadials (MIS 19-s1–s4) and interstadials (MIS 19-i1–i4) follows Haneda et al. (2020b) but with modified labeling and MIS 19a benthic isotope oscillations (MIS 19a-*o*1 to MIS 19a-*o*4) following Head (2021). Blue arrow marks the suggested position of a Younger Dryas-type cooling event according to Suganuma et al. (2018)

**Fig. 19:**
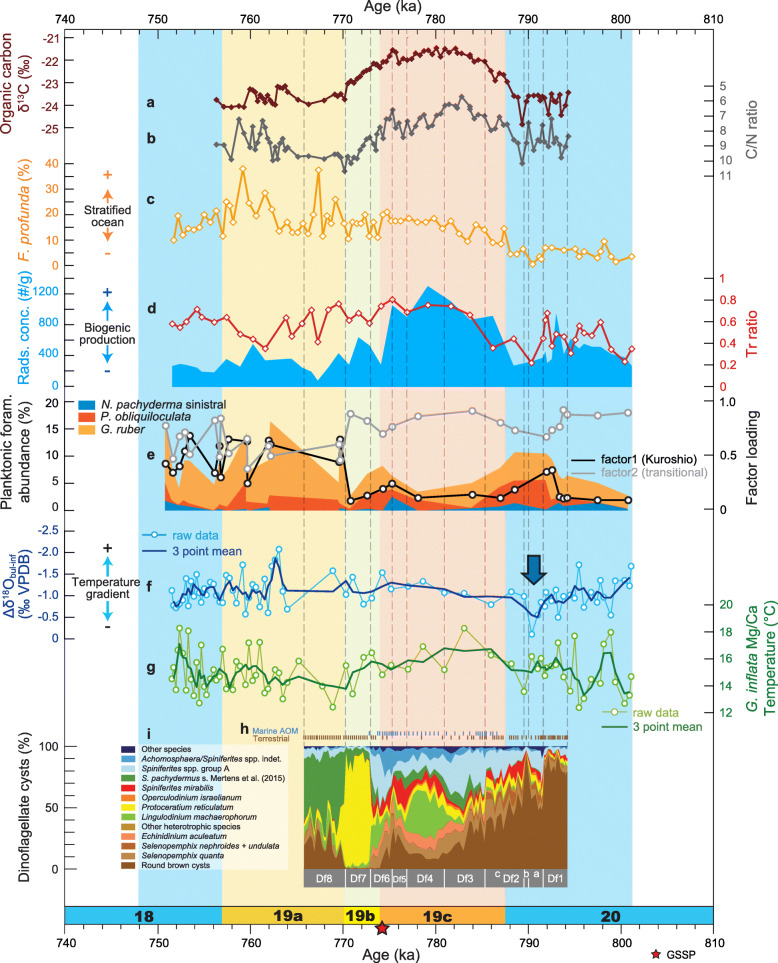
Dinoflagellate cyst record (this study) compared with paleoclimatic and paleoceanographic records through MIS 20 to MIS 18 from the Chiba composite section (CbCS) (from Suganuma et al. 2018, in press). **a** Organic carbon stable isotope (δ^13^C_org_) and **b** C/N ratio (= total organic carbon [TOC]/total nitrogen [TN]) for bulk samples (Izumi et al. 2021). **c** Relative abundance of nannofossil *F*. *profunda* (Kameo et al. 2020). **d** Blue shaded area: Radiolarian concentrations (proxy for biological production); red line: Tr values (sea-surface temperature index based on warm- and cold-water radiolarian species). **e** Relative abundance of planktonic foraminifera and results of factor analysis. **f** ∆δ^18^O_*G*. *bulloides*–*G*. *inflata* (bul-inf)_, corresponding to the temperature gradient (hence stratification) between surface and subsurface waters. **g** Mg/Ca paleotemperature for *G*. *inflata* (T_inf_) considered to represent the subsurface (> 100 m) winter temperature (Kubota et al. 2021). **h** Dominant palynofacies component (brown line, terrestrial; blue line, marine amorphous organic matter [AOM]) from Fig. 16. **i** Dinoflagellate cyst synthetic diagram showing most abundant taxa and local assemblage biozonation (this study). Blue arrow marks the suggested position of a Younger Dryas-type cooling event according to Suganuma et al. (2018)

Numerous proxies have been used to reconstruct the paleoenvironmental history of the Chiba composite section, including foraminiferal isotopes, pollen, and organic geochemistry at very high temporal resolution as discussed in detail in Suganuma et al. (2018, in press). These highly resolved records are integrated and compared with the marine palynological data (Figs. 17, 18, and 19) below.

### Dinoflagellate cyst assemblage biozone Df1 (794.21–791.72 ka), upper MIS 20

This biozone reflects cold and relatively nutrient-rich subarctic water masses based on the overwhelming dominance of round brown cysts (76–93%). The Subarctic Front would therefore have been south of the Chiba section. There are no close modern analogues of this cyst association, although high abundances of round brown cysts up to ~ 60% are found in the northernmost North Pacific today (Bonnet et al. 2012). Differences with these modern assemblages include the lower abundances of *Nematosphaerposis labyrinthus* and *Pyxidinopsis reticulata*, perhaps implying lower salinities than at present in the northernmost North Pacific, and apparent absence of *Spiniferites elongatus* which may suggest somewhat warmer conditions for the Chiba composite section. Today, the dominance of round brown cysts is generally associated with high biological productivity (Zonneveld et al. 2013), but cyst concentrations are not elevated in biozone Df1. High sedimentation rates of 1.23 m/kyr (Suganuma et al., in press) will have had a diluting effect during biozone Df1, but productivity does not seem to be have been especially elevated unless round brown cysts are underrepresented significantly through preservational loss.

During this time, insolation for June was increasing (Fig. 17). Other marine proxies in general reflect cool water masses, although Mg/Ca values for *Globorotalia inflata* indicate rather high winter–spring temperatures of ~ 15.5 °C at > 100 m depth (Fig. 19). The pollen record shows ameliorating climatic conditions on land with an increase in broadleaved deciduous species already by 796 ka. Biozone Df1 coincides with the lower third of pollen subzone 1b of Suganuma et al. (2018) which represents subarctic coniferous and deciduous broadleaved mixed forest (Fig. 17). The dominance of terrestrially derived organic matter can be explained by transport from a partially exposed shelf owing to the lowered global sea level, but also apparently by relatively low marine productivity as discussed above and inferred from other marine indicators including low radiolarian concentrations and more negative δ^13^C_org_ and higher Ca/Ti ratios (Suganuma et al., in press; Izumi et al. 2021; Fig. 19).

In general, the progressive decrease in round brown cysts from biozone Df1 through Df3, which represents the transition from glacial MIS 20 to interglacial MIS 19, signals the decreasing influence of cold subarctic surface waters. This trend closely matches that of increasing temperate tree pollen (*Quercus* subgenus *Lepidobalanus* pollen and of total deciduous broadleaved plants; Fig. 17) which signals increasingly warm conditions on land. It also matches the foraminiferal δ^18^O stratigraphy which shows a general trend of decreasing values through this interval (Fig. 18b).

### Dinoflagellate cyst assemblage biozone Df2 (791.55–785.47 ka), upper MIS 20–lower MIS 19c

This biozone encompasses Termination IX and is divided into three sub-biozones.

### Sub-biozone Df2a (791.55–790.32 ka), upper MIS 20

An abrupt decline in round brown cysts (85–52%) and rise in *Spiniferites* spp. group A at the beginning of this zone suggests a reduction in the influence of subarctic water masses and therefore a rise in SSTs. A small increase in *Operculodinium israelianum*, a mostly tropical–subtropical species (Zonneveld et al. 2013), to ~ 3% is consistent with this suggested rise in SST.

Within the pollen record, however, a discrete warming event is not recognized, and within the *Globigerina bulloides* δ^18^O record sub-biozone Df2a is represented by higher values that have been tentatively attributed to a Younger-Dryas-like cooling event (Suganuma et al. 2018; Figs. 18 and 19). One possible explanation for this discrepancy is that because round brown cysts are particularly susceptible to oxidation on the sea floor (Hopkins and McCarthy 2002), a Younger-Dryas-like cooling event might have invigorated bottom-water oxygenation and caused the selective loss of some round brown cysts. Nonetheless, Mg/Ca values for *Globorotalia inflata* indicate rather stable winter–spring subsurface (> 100 m) temperatures at around 15.5 °C, at odds with the near-surface (< 50 m) *Globigerina bulloides* δ^18^O record.

### Sub-biozone Df2b (789.76 ka), upper MIS 20

A single sample containing abundant round brown cysts (92%) might reflect a brief southward return of the Subarctic Front and hence represent a cooling event.

Brief cooling is consistent with single δ^18^O and Mg/Ca data points for *Globorotalia inflata*, and this interval is close to single-point deflections in the total organic carbon/total nitrogen (C/N) and δ^13^C_org_ record for bulk samples (Figs. 18 and 19). Higher resolution multi-proxy sampling is needed to substantiate this possible event.

### Sub-biozone Df2c (789.31–785.47 ka), upper MIS 20–lower MIS 19c

At the base of this sub-biozone, a sharp drop in the abundance of round brown cysts and a rise in *Spiniferites mirabilis* to 16% indicates a significant rise in sea-surface temperatures. This temperate to tropical species has its distributional limits today approximately following the subtropical fronts on both hemispheres (Zonneveld et al. 2013; de Vernal et al. 2020). Abundances greater than 15% occur typically where summer SSTs are 15 °C or more, and summer SSS exceed ~ 32 psu. It occurs in coastal and open ocean environments and can tolerate reduced salinities and both oligotrophic and nutrient-elevated conditions (Zonneveld et al. 2013). In the northern North Pacific today, *Spiniferites mirabilis* is associated only with more southerly sites under the influence of the Kuroshio Extension (Bonnet et al. 2012). This implies that the Subarctic Front moved rapidly north of the Chiba composite section during the beginning of sub-biozone Df2c at ~ 789.31 ka.

The interpreted rapid warming at the start of sub-biozone Df2c (789.31 ka) is consistent with most marine proxies including the benthic and planktonic foraminiferal δ^18^O records (Figs. 18 and 19), and also with increasing pollen of deciduous broad-leaved trees through this subzone (Fig. 17). The northward shift of the Subarctic Front at ~ 789.31 ka predates the end of MIS 20 and also the peak in June insolation by about 2000 years (Fig. 17). Changes in palynofacies, with marine AOM becoming more common from ~ 787.28 ka and then mostly abundant to dominant from 786.60 ka, precisely follow increases in δ^13^C_org_ and decreases in C/N values (Suganuma et al., in press; Izumi et al. 2021; Fig. 19). These changes occur just after the start of MIS 19c and also coincide precisely with a sharp reduction in values of *G*. *bulloides* δ^18^O at 787 ka. They signify a lower input of terrestrial material and/or higher biogenic productivity at a time of rapid sea-surface warming, with the combination of sea-level rise and warmer waters in the earliest part of MIS 19c driving this change. Increasing marine AOM from 787.28 ka seems to reflect mostly a rise in biogenic productivity which is indicated by a rise in radiolarian concentrations in the upper part of this zone (Suganuma et al. 2018, in press).

### Dinoflagellate cyst assemblage biozone Df3 (785.20–781.20 ka), MIS 19c

This biozone Df3 is marked by generally sustained abundances (1.6–15%) of *Spiniferites pachydermus* sensu Mertens et al. (2015), a species occurring in modern sediments of İzmir Bay, eastern Aegean Sea, Mediterranean where the waters are eutrophic, summer SSTs are ~ 25–27 °C, and summer SSSs are ~ 33–39 psu for the inner bay (Mertens et al. 2015; Sayın et al. 2006). Although there are uncertainties regarding the identity of this distinctive undescribed species, it has likely been recorded at least in part from modern sediments under the name *Spiniferites pachydermus*, a coastal to open marine species considered to have a temperate to tropical distribution, with abundances greater than 5% when summer SSTs exceed ~ 18 °C and summer SSSs exceed ~ 33 psu (Zonneveld et al. 2013). It is restricted to fully marine environments where mesotrophic to eutrophic conditions occur including upwelling areas (Zonneveld et al. 2013). *Spiniferites pachydermus* sensu Mertens et al. (2015) has possibly been reported from Holocene shallow-marine sediments of the Tsushima Islands, west Japan (Matsuoka 1994, as *Spiniferites* cf. *bentorii*), which are under the influence of the Kuroshio/Tsushima Warm currents. *Spiniferites* spp. group A is an abundant (20–37%) constituent of biozone Df3, and as with most other members of this genus, represents a neritic component. Elevated abundances of the heterotroph *Echinidinium aculeatum* (0.3–10%) in this biozone are also significant. This species has a mesotrophic/ eutrophic temperate to tropical modern global distribution, being confined to regions with unstratified upper waters (Zonneveld et al. 2013).

The tentative estimate of summer SST exceeding ~ 18 °C based on *Spiniferites pachydermus* (possibly in the sense of Mertens et al. 2015) is broadly consistent with subsurface (> 100 m) winter temperatures of 15–18 °C based on *Globorotalia inflata* Mg/Ca data for biozone Df3. The start of biozone Df3 coincides with the onset of full interglacial conditions on land, as registered by high abundances of deciduous tree pollen including *Quercus* subgenus *Lepidobalanus* and the sustained presence of evergreen tree pollen (Fig. 17). All marine proxies are consistent with sea-surface temperatures that are among the warmest for the Chiba composite section. The general co-dominance or alternating dominance of marine AOM with terrestrial organic matter throughout this zone is reflected also in the δ^13^C_org_ and C/N values (Suganuma et al., in press; Izumi et al. 2021; Fig. 19a, b) and presumably relates to high biological productivity judging from the radiolarian concentrations (Fig. 19d).

### Dinoflagellate cyst assemblage biozone Df4 (780.74–777.13 ka), MIS 19c

*Lingulodinium machaerophorum* (17–39%) characterizes this biozone. This is a temperate to tropical euryhaline species particularly abundant today in (1) estuarine environments with increased nutrients and lowered salinities (e.g., Wall et al. 1977; Morzadec-Kerfourn 1977; Dale et al. 1999), (2) bays that have experienced eutrophication (Dale and Fjellså 1994; Dale et al. 1999; Matsuoka 1999; Shin et al. 2010), (3) below river discharge plumes (Zonneveld et al. 2013), and (4) areas affected by elevated nutrient levels arising from diffuse upwelling (Dale 1996; Zonneveld et al. 2013). The thermophilic *Spiniferites mirabilis* remains at significant levels (2.9–16%). *Spiniferites mirabilis* today is mostly distributed within the subtropical fronts of both hemispheres. It is considered a temperate to tropical species, and can be associated with eutrophic environments (Zonneveld et al. 2013). The tropical–subtropical *Operculodinium israelianum* although not common (≤ 3.3%) has its highest occurrence in this zone. This biozone therefore signals an increase in nutrient input to the surface waters, with a possible lowering of salinity. It might be related to a southward shift in the position of the Kuroshio Extension or an increase in its intensity that would induce a diffuse upwelling of nutrient-elevated waters.

Full interglacial conditions on land and warm sea-surface conditions continue through biozone Df4 as reflected in all proxies. However, a strong upwelling signal does not appear to be reflected in the other marine proxies. Relatively high abundances of the coccolith *Florisphaera profunda* (Fig. 19c) indeed suggests moderate water column stratification, although not necessarily at the time of year when *Lingulodinium machaerophorum* would have been blooming. Relatively high abundances of *Echinidinium aculeatum* (6.8–10%) throughout this zone suggest weak stratification, as noted above (Zonneveld et al. 2013). Relatively weak stratification is also reflected in the difference between benthic and surface dwelling (*G*. *bulloides*) foraminiferal δ^18^O values at this time (fig. 3d of Haneda et al. 2020b). Slightly higher benthic and planktonic foraminiferal δ^18^O values (Fig. 18b) in the middle of this zone where *Lingulodinium machaerophorum* reaches highest abundances (39%) corroborate the interpretation that the Kuroshio Extension may have shifted southwards, resulting in diffuse upwelling. The dominance of marine AOM through this biozone is consistent with relatively high marine organic productivity, as suggested by continued high δ^13^C_org_ values and radiolarian concentrations (Fig. 19a, d).

### Dinoflagellate cyst assemblage biozone Df5 (776.68–775.44 ka), MIS 19c

This biozone records a sharp decline in *Lingulodinium machaerophorum* (to 5.2%) and a rise in *Spiniferites mirabilis* to its highest abundances (21%) in the Chiba composite section. *Spiniferites mirabilis* has abundances greater than 20% today mostly where summer SSTs are 16 °C or more, and summer SSS exceed ~ 32 psu (Zonneveld et al. 2013). *Spiniferites pachydermus* sensu Mertens et al. (2015) has values of 1.1–10%, and species of the oceanic genus *Impagidinium* become somewhat more abundant, rising to 1.8%. The equatorial to temperate *Echinidinium aculeatum* has a peak abundance of 12% in this zone, implying continued weak surface water stratification. *Tuberculodinium vancampoae*, which rises to 2.3%, is a temperate to tropical species found in coastal embayments around Japan today. It mostly occurs in central and southern areas of Japan (Zonneveld et al. 2013) but extends to northeastern Honshu (Matsuoka 1976). It has been reported from the mid-Pleistocene of Osaka Bay to occasionally dominate or co-dominate assemblages (Harada 1984). It presumably represents transport from the shelf. The assemblage composition in general therefore indicates warm, weakly stratified surface waters, which accords with benthic foraminiferal δ^18^O values that are at their lowest, and planktonic δ^18^O values that become increasingly low through this biozone (Fig. 18b). The pollen record reflects full interglacial conditions on land, and most marine proxies point to continuing warm SSTs. A northward shift of the Kuroshio Extension best explains this combination of observations. The continued dominance of marine AOM is again consistent with relatively high marine organic productivity based on high δ^13^C_org_ values and radiolarian concentrations (Fig. 19a, d).

### Dinoflagellate cyst assemblage biozone Df6 (775.21–773.07 ka), MIS 19c–b

This biozone represents a brief increase in *Lingulodinium machaerophorum*, reaching 31% in its upper part, implying the return of diffuse nutrient input to the surface waters, as inferred for biozone Df4. The presence of *Echinidinium aculeatum* (2.6–8.4%) is consistent with weak surface water stratification. Significant values of *Spiniferites mirabilis* (4.1–20%) and of *Spiniferites pachydermus* sensu Mertens et al. (2015) rising to 17% near the top of this zone indicate continued warm SSTs, as does *Tuberculodinium vancampoae* which reaches its maximum of 4.4% in this zone. The abundance peak of the extinct acritarch *Nanobarbophora walldalei* (up to 5.0% of the total in-situ dinoflagellate cysts) is consistent with this interpretation, as this species is associated with tropical to warm/mild-temperate paleoenvironments (Head *in* Head and Westphal 1999; Head 2003) and is associated with warmer MISs in the Mediterranean (as Acritarch sp. B in Versteegh 1994, 1995; Versteegh and Zonneveld 1994). *Selenopemphix quanta* reaches its highest abundance (21%) in this zone. This species has a broad temperature tolerance and occurs from tropical to subpolar regions today but is most abundant in upwelling regions and at oceanic fronts where nutrient levels are elevated (Zonneveld et al. 2013). On balance, biozone Df6 particularly in its latter half suggests a slight southward shift of the Kuroshio Extension, bringing mixed waters of the KOIZ over the site of deposition, and hence somewhat elevated nutrient levels. It may represent a transitional state between biozones Df5 and Df7.

This interpretation would imply somewhat cooler waters for this biozone, particularly during the latter half. Biozone Df6 spans the MIS 19c–b boundary which is marked by progressively higher benthic foraminiferal δ^18^O values. The base of this biozone corresponds to the termination of full interglacial conditions as determined by pollen analysis, and a decline in deciduous tree pollen in the lower part of biozone Df6 reflects cooling on land, which is followed by a warming in the upper part of the biozone based on the MAT reconstruction (Fig. 17). Planktonic foraminiferal δ^18^O values in contrast are very low except at the top of this biozone where they become markedly higher (Fig. 18b).

In general, marine proxies reflect transitional conditions through this biozone. SSTs nonetheless remained warm as corroborated by values exceeding ~ 15 °C based on Mg/Ca data for *Globorotalia inflata* (Fig. 19g), but likely dropped near the top of this biozone judging from the radiolarian Tr ratio. Low percentages of the calcareous nannofossil *F*. *profunda* in the latter part of this zone would seem to confirm reduced ocean stratification at this time. A significant shift from marine AOM-dominated palynofacies in the lower part of the biozone to a mostly terrestrially dominated palynofacies from 773.52 ka near the top of the biozone is mirrored by declining δ^13^C_org_ and increasing C/N values (Fig. 19a, b). It appears to represent a sharp decline in biological productivity based on the reduced concentration of radiolarians (Fig. 19d). It also corresponds to a sharp drop in SST as inferred from higher planktonic foraminiferal δ^18^O values, this signaling the onset of stadial 1 (MIS 19-s1) within the earlier part of MIS 19b (Fig. 18).

### Dinoflagellate cyst assemblage biozone Df7 (772.85–770.37 ka), MIS 19b

This biozone represents the most abrupt change in the examined interval of the Chiba composite section, with cysts of *Protoceratium reticulatum* (73–93%) rising rapidly in abundance at the base of the zone, dominating throughout, and falling abruptly at the end of this biozone. The only other relatively common species is the thermophilic *Spiniferites pachydermus* sensu Mertens et al. (2015) (3.7–18%). *Protoceratium reticulatum* is a potentially yessotoxin-producing (Liu et al. 2017; Wang et al. 2019), bloom-forming opportunistic dinoflagellate found in Japanese waters today (e.g., Koike et al. 2006). It is a cosmopolitan species whose cysts, known in the literature also as *Operculodinium centrocarpum* sensu Wall and Dale (1966), can dominate today at high latitudes including the Okhotsk Sea (Bonnet et al. 2012) and off Hachinohe, northeastern Japan, which is under the influence of the Tsugaru Warm Current (Matsuoka 1976) and Oyashio Current. It also dominates the autotrophic component of modern cyst assemblages from Akkeshi Bay, eastern Hokkaido, which is also strongly influenced by the Oyashio Current (Matsuoka 1987). Its global modern distribution shows an affinity for mesotrophic to eutrophic and cold to temperate environments (Bonnet et al. 2012), and it appears well adapted to unstable conditions where oceanic and neritic waters mix (Dale 1996). This is seen in the North Atlantic where it serves as an indicator for the North Atlantic Current both in modern assemblages (Harland 1983; Rochon et al. 1999; Zonneveld et al. 2013) and in Plio-Pleistocene records (e.g., De Schepper et al. 2009, 2013; Hennissen et al. 2014, 2017). Unusually, it can be abundant in both neritic and oceanic settings, and this may be attributable to its unusual life cycle (Salgado et al. 2017) that can sustain productivity in oceanic waters where sufficient nutrients exist. This species appears to represent summer sea-surface conditions, given that within the surface waters of Okkirai Bay, northeastern Japan, motile cells of *Protoceratium reticulatum* were observed only from mid-June to late August, with peak abundances recorded from late June to late July. The highest density of cells was observed in the surface layer (0–5 m), and maximum abundance appeared to be correlated to periods of lowered surface water salinity due to rainfall (Koike et al. 2006). Similarly, in the northern Yellow Sea of China, motile cells of *Protoceratium reticulatum* were found to be largely restricted to the months of April, May, and June, and the cysts most common in the sediment from March to August (Liu et al. 2017). The cysts of this species form when nutrient levels decline, and the cysts themselves have an obligatory dormancy period of ~ 4 months. The cysts are clearly important to the ecology of this species, and appear to represent an overwintering strategy when conditions are unfavorable for population growth (Salgado et al. 2017). The dominance of *Protoceratium reticulatum* cysts in biozone Df7 is therefore interpreted to reflect cooler, nutrient-elevated and unstable spring–summer surface waters under the influence of the KOIZ, brought about by a southward shift of the Kuroshio Extension. It may be noted that this species has been reported from the mid-Pleistocene of Osaka Bay, but while abundant in some samples (Harada 1984), it does not attain the exceptional abundances that characterize biozone Df7.

Biozone Df7 spans the upper two-thirds of MIS 19b, this substage representing the glacial inception that terminates the interglacial conditions of MIS 19c. This biozone includes much of stadial 1 and the lower half of interstadial 1 (Fig. 18; Head 2021). Its onset is marked by an abrupt shift to higher planktonic foraminiferal δ^18^O values (Fig. 18b), implying cooler surface waters, and occurs within a trend of increasing benthic foraminiferal δ^18^O values through MIS 19b and specifically at a minor increase. However, other marine proxies do not record pronounced cooling, although factor analysis of planktonic foraminiferal assemblages shows a slight increase in transitional (cooler) waters during biozone Df7 and a slight decrease in the influence of the Kuroshio Current (Fig. 19e). Radiolarian Tr values also indicate somewhat cooler waters. Lower abundances of the coccolith *Florisphaera profunda* (Fig. 19c) indicate reduced water column stratification consistent with the interpretation of unstable conditions. The pollen record similarly does not show pronounced changes with the exception of an increased abundance of the temperate conifer *Cryptomeria* during biozones Df6 and 7 which possibly indicates gradual cooling (Suganuma et al. 2018).

The highest cyst concentrations (21,639–317,932 cysts/gram) are recorded in biozone Df7, implying enhanced biological productivity consistent with the influence of the nutrient-rich KOIZ. In contrast, however, δ^13^C_org_ and C/N values continue their declining/increasing trends respectively through this biozone, and marine AOM is subordinate to plant tissues and terrestrial sporomorphs in all but the lowest sample of this biozone. In addition, radiolarian concentrations as a measure of biogenic production show much lower values than for MIS 19c. It is possible, therefore, that high dinoflagellate cyst concentrations reflect productivity limited to the spring–summer months and are not representative of annual biological production.

The abrupt rise in *Protoceratium reticulatum* cysts at the inception of biozone Df7 occurs approximately across two samples separated by 20 cm (TB2-26 at 0.95 m and TB2-24 at 1.15 m above the Byk-E bed) and suggests a change to cooler summer SSTs within ~ 300 years. This pronounced paleoceanographic shift occurs within the directional transition zone (0.25–1.95 m) of the Matuyama–Brunhes paleomagnetic reversal, and is indeed close to its midpoint which is astronomically dated at 772.9 ka. A connection between a collapse in the Earth’s magnetic field intensity, as occurs during a reversal, and increased atmospheric penetration of galactic cosmic rays leading to more extensive cloud cover and ultimately global cooling, has been proposed frequently in the literature (Kitaba et al. 2017 and references therein) but remains open to debate. The timing of the onset of biozone Df7 does not however coincide with the start of field intensity decline, as indicated by the ^10^Be and paleointensity records, which is progressive and begins earlier than the directional transition zone (Okada et al. 2017; Simon et al. 2019; Haneda et al. 2020a). Moreover, *Protoceratium reticulatum* cyst abundance at the end of this biozone drops almost as rapidly as it begins without any obvious change in the field intensity, which remains low until ~ 760 ka (Simon et al. 2019; Haneda et al. 2020a).

### Dinoflagellate cyst assemblage biozone Df8 (770.14–765.80 ka), MIS 19a

A steep decline in *Protoceratium reticulatum* cysts from 93% at the top of biozone Df7 to 47% at the base of biozone Df8 and then to a minimum of 2%, and corresponding rise to dominance of *Spiniferites pachydermus* sensu Mertens et al. (2015) with abundances of 38–69%, represents the abrupt transition to a unique association within the Chiba record. The latter species is a known thermophile and, as noted above, probably well adapted to fully marine environments when mesotrophic to eutrophic conditions prevail. Moderate surface-water nutrient levels are also reflected by a relative increase in heterotrophic dinoflagellate cysts, including round brown cysts (2.3–38%) and *Selenopemphix quanta* (0.3–13%).

Cyst concentrations (2459 to 19,205 cysts/g), although representing a significant decline from peak values during biozone Df7, remain higher than before that time. In contrast, marine AOM is rare, and consistently low δ^13^C_org_ values, high C/N ratios, and low concentrations of radiolarians (Fig. 19) all reflect low overall biological productivity for this biozone.

The apparent contradiction between moderate surface-water nutrient levels and low biological productivity may be linked to water-column stratification. Elevated abundances of the coccolith *Florisphaera profunda* indicate pronounced water column stratification (Fig. 19c), and the near absence of the extant *Echinidinium aculeatum* which today is confined to regions with unstratified upper waters (Zonneveld et al. 2013) is consistent with stratification throughout this biozone.

An abrupt shift to lower values in both the planktonic and especially benthic foraminiferal δ^18^O values signifies warming at the onset of biozone Df8 and defines the base of MIS 19a. While the onset of biozone Df8 aligns precisely with that of MIS 19a-*o*1, it occurs midway through the interstadial MIS 19-i1 as characterized by the planktonic foraminiferal δ^18^O record (Fig. 18). This discrepancy is perhaps related to seasonal differences in the proxies examined: the foraminifera registering winter–spring conditions and at least some dinoflagellates recording the spring–fall. There are no substantial changes in assemblage composition through biozone Df8. However, both planktonic and benthic foraminiferal δ^18^O values are higher in its upper half, from ~ 768 ka onwards, corresponding to the cooler conditions of stadial 2 within MIS 19a (Fig. 18), and this is perhaps reflected in the lower cyst concentrations recorded in the second half of the biozone.

The increase in June insolation at this time (Figs. 17 and 18a) may have contributed to surface water stratification. The abundance of *Spiniferites pachydermus* sensu Mertens et al. (2015) therefore possibly relates to summer water-column stratification.

Hence, biozone Df8 is interpreted to mark the establishment of warm, stratified, nutrient-elevated, surface water conditions that persisted to the top of the examined interval. The Kuroshio Extension Front is interpreted to have shifted northwards at the beginning of this zone, causing the intrusion of the southern margin of the KOIZ.

This interpretation is corroborated by varimax factor loadings and reconstructed SSTs of planktonic foraminiferal assemblages which show at ~ 770 ka an abrupt rise in the influence of the warm Kuroshio Current, and a concomitant decline in (cool) transitional waters. The pollen record for biozone Df8 is at low stratigraphic resolution, but increasing values of the temperate conifers *Tsuga* and *Picea* (Fig. 17) indicate a cooler climate on land than for biozone Df7.

Suganuma et al. (2018) had previously proposed a northward shift of the Kuroshio Extension at this time, postdating the termination of full interglacial conditions on land by ~ 5 kyrs. These authors proposed that relatively high winter insolation at 50° N at this time might have contributed to a weakening of the Siberian High, a winter phenomenon, and hence weakening of the East Asian Winter Monsoon (EAWM). This will have allowed the westerly jet, which influences the position of the Kuroshio Current, to take a more northerly path in winter. Suganuma et al. (2018) provided two lines of evidence for this. Firstly, grain-size variations in loess-paleosol successions on the Chinese Loess Plateau indicate a weak EAWM for up to 20,000 years after the end of fully interglacial conditions (Sun et al. 2006; Hao et al. 2012). Secondly, from the Chiba composite section itself, a slight increase in the differential between the δ^18^O of the surface-dwelling foraminifera *G*. *bulloides* and subsurface *G*. *inflata* indicates an increase in water column stratification during the cold seasons because these two species are abundant at different depths off the Japanese archipelago today in winter (Suganuma et al. 2018). Therefore, increased water column stratification during winter, representing decreased wind mixing, is consistent with a weaker EAWM. Sea-surface conditions were warm as indicated by the thermophilic *Spiniferites pachydermus* sensu Mertens et al. (2015), but the season(s) of maximum growth and cyst production are not known for this species. A summer northward shift of the Kuroshio Extension Front might, however, be needed to explain the abundance of this species. Evidence from the Chinese Loess Plateau provides a pattern of weakening summer monsoons during this time (Sun et al. 2006) which would have reduced wind stress over the ocean. This together with the peak in June insolation at this time (Figs. 17 and 18) may therefore provide a mechanism for water-column stratification and elevated sea-surface temperatures during the spring–summer months. This raises the possibility that the westerly jet, which influences the latitude of the Kuroshio Extension, took a more northerly path in summer, as well as in winter, relative to summer conditions during biozone Df7. More information is needed on the seasonality of *Spiniferites pachydermus* sensu Mertens et al. (2015) to clarify this possibility.

## Conclusions

This is the first detailed dinoflagellate cyst study of the Pleistocene from the Pacific margin of the Japanese islands. Using closely spaced (10 or 20 cm) samples from the highly constrained adjacent Chiba and Yoro-Tabuchi sections of the Chiba composite section, it establishes a detailed paleoceanographic reconstruction (794.21 to 765.80 ka; late MIS 20 to mid-MIS 19a) across the Early–Middle Pleistocene Subseries (Calabrian–Chibanian Stage) boundary at the type locality. The location is presently close to the confluence of the warm Kuroshio and cold Oyashio currents and is therefore well positioned to explore their relative influences over time, especially during MIS 19 when conditions were similar to the present interglacial. The dinoflagellate cyst record reveals previously undocumented detail regarding surface-water instability through MIS 19, including the response of dinoflagellates to Termination IX, to full interglacial conditions during MIS 19c, the arrival of cooler conditions during MIS 19b, and a return to warmer conditions at the beginning of MIS 19a during interstadial MIS 19-i1. The dinoflagellate cyst assemblages are overwhelmingly dominated by neritic taxa, and preservation varies from good to moderate. The palynofacies is characterized by common to dominant terrestrial material, and it is therefore assumed that dinoflagellate cyst assemblages reflect changes across the continental shelf and not simply oceanic waters above the continental slope on which the sediments were deposited. The dominance of marine AOM particularly between 786.60 and 772.85 ka, representing most of MIS 19c and the earliest part of MIS 19b, closely follows the high-resolution records for δ^13^C_org_ and C/N (Suganuma et al., in press; Izumi et al. 2021; Fig. 19) and corroborates the interpretation based on elevated concentrations of radiolarians (Fig. 19) that biological production was enhanced through this interval. The dinoflagellate cyst record is subdivided into eight assemblage biozones.

Dinoflagellate cyst assemblage biozone Df1 (794.21–791.72 ka, upper MIS 20) is dominated by heterotrophic round brown cysts and reflects cold and relatively nutrient-rich subarctic waters, although presumably not fully glacial conditions.

Sub-biozone Df2a (791.55–790.32 ka, upper MIS 20) is characterized by a reduction in round brown cysts that suggests a reduced influence of subarctic water masses and therefore a rise in SSTs. However, preservational factors cannot be excluded as some marine proxies suggest a Younger-Dryas-like cooling event during this interval.

Sub-biozone Df2b (789.76 ka, upper MIS 20) is represented by a single sample dominated by round brown cysts and which might represent a brief southward return of the Subarctic Front, although further sampling through this interval is needed to confirm a possible cooling event.

Sub-biozone Df2c (789.31–785.47 ka, upper MIS 20–lower MIS 19c) is marked by a sharp drop in the abundance of round brown cysts and a rise in *Spiniferites mirabilis* indicating a significant rise in sea-surface temperatures and rapid retreat of the Subarctic Front at ~ 789.31 ka, predating the end of MIS 20 and also the peak in June insolation by ~ 2000 years. A relative increase in marine AOM from ~ 787 ka is consistent with a rise in biogenic productivity.

Dinoflagellate cyst assemblage biozone Df3 (785.20–781.20 ka, MIS 19c) includes a sustained presence of the thermophilic *Spiniferites pachydermus* sensu Mertens et al. (2015) and represents warm and relatively unstratified surface waters with elevated nutrient levels. Its inception coincides with the onset of full interglacial conditions on land. Other marine proxies give sea-surface temperatures that are among the warmest for the Chiba composite section.

Dinoflagellate cyst assemblage biozone Df4 (780.74–777.13 ka, MIS 19c), characterized by relatively high values of *Echinidinium aculeatum* and abundant *Lingulodinium machaerophorum*, signals elevated nutrient levels and perhaps a lowering of salinity, and appears to represent diffuse upwelling possibly related to a southward shift in the position of the Kuroshio Extension or an increase in its intensity. The dominance of marine AOM through this zone is consistent with relatively high marine organic productivity as reflected by other proxies.

Dinoflagellate cyst assemblage biozone Df5 (776.68–775.44 ka, MIS 19c) is marked by a steep decline in *Lingulodinium machaerophorum* and rise in the abundance of the thermophilic *Spiniferites mirabilis* and *Echinidinium aculeatum* to their highest values implying continued warm, weakly stratified surface waters with reduced nutrient levels, and suggesting a northward shift of the Kuroshio Extension.

Dinoflagellate cyst assemblage biozone Df6 (775.21–773.07 ka, MIS 19c–b) marks a brief return to an increased abundance of *Lingulodinium machaerophorum* and suggests, particularly during its latter half, a slight southward shift of the Kuroshio Extension. It may represent a transitional state between biozones Df5 and Df7. Although the base of this biozone corresponds to the termination of full interglacial conditions as determined by pollen analysis, and the termination of MIS 19c falls with the middle of this biozone, significant cooling is not registered in the dinoflagellate cyst record. A shift from marine AOM-dominated to a mostly terrestrially dominated palynofacies at 773.52 ka appears to reflect a sharp decline in biological productivity and signals the onset of MIS 19-s1 (stadial 1) within the earlier part of MIS 19b.

Dinoflagellate cyst assemblage biozone Df7 (772.85–770.37 ka, MIS 19b) represents an abrupt (within ~ 300 years) and sustained dominance of *Protoceratium reticulatum* cysts accompanied by a one- or two-order magnitude increase in cyst concentrations, signaling the rapid onset of unstable, cooler, and biologically productive surface waters during the spring–summer months. This is interpreted to mark the influence of the Kuroshio–Oyashio Interfrontal Zone resulting from a southward shift in the Kuroshio Extension. This biozone corresponds to the upper two thirds of MIS 19b and its onset broadly coincides with the glacial inception terminating the interglacial conditions of MIS 19c. The abrupt rise in *Protoceratium reticulatum* cysts defining the base of biozone Df7 also serves as an unambiguous local ecostratigraphic marker for the Chibanian Stage GSSP which occurs just 1.15 m (= 1300 years) below it. This rise also coincides with the directional midpoint of the Matuyama–Brunhes boundary in the Chiba section (Haneda et al. 2020a).

Dinoflagellate cyst assemblage biozone Df8 (770.14–765.80 ka, MIS 19a) represents an abrupt replacement of *Protoceratium reticulatum* cysts with the thermophilic *Spiniferites pachydermus* sensu Mertens et al. (2015), marking the intrusion of warmer although still nutrient enriched surface waters. This is interpreted to represent a northward shift of the Kuroshio Extension Front and exposure to the southern margin of the Kuroshio–Oyashio Interfrontal Zone. Pronounced water column stratification seems best to explain the composition of assemblages in this biozone including the near absence of *Echinidinium aculeatum*. Ecological information on the season(s) of maximum growth and cyst production is not available for *Spiniferites pachydermus* sensu Mertens et al. (2015), but if its abundance represents a summer signal then this coincides with a peak in June insolation. These conditions imply a northward shift of the westerly jet in summer which would permit the Kuroshio Current to reach higher latitudes than previously. A simultaneous weakening of the East Asian Summer Monsoon would also explain water-column stratification at this time.

## Supplementary Information

**Additional file 1.** Raw data including counts of all dinoflagellate cysts and other palynomorphs.

## Data Availability

Data supporting the results reported in this article are provided in Additional file 1.

## Abbreviations

AOM: Amorphous organic matter
Byk-E: Ontake-Byakubi-E tephra bed
EASM: East Asian Summer Monsoon
EAWM: East Asian Winter Monsoon
KOIZ: Kuroshio–Oyashio Interfrontal Zone
GSSP: Global Boundary Section and Point
MIS: Marine Isotope Stage
SSS: Sea-surface salinity
SST: Sea-surface temperature
